# The glucocorticoid receptor as a master regulator of the Müller cell response to diabetic conditions in mice

**DOI:** 10.1186/s12974-024-03021-x

**Published:** 2024-01-25

**Authors:** Anna M. Pfaller, Lew Kaplan, Madalena Carido, Felix Grassmann, Nundehui Díaz-Lezama, Farhad Ghaseminejad, Kirsten A. Wunderlich, Sarah Glänzer, Oliver Bludau, Thomas Pannicke, Bernhard H. F. Weber, Susanne F. Koch, Boyan Bonev, Stefanie M. Hauck, Antje Grosche

**Affiliations:** 1 Department of Physiological Genomics, Biomedical Center-BMC, Ludwig-Maximilians-Universität München, Planegg-Martinsried, Germany; 2 Helmholtz Pioneer Campus, Helmholtz Zentrum München, German Research Center for Environmental Health, Neuherberg, Germany; 3 Institute of Clinical Human Genetics, University Hospital Regensburg, Regensburg, Germany; 4 Institute for Clinical Research and Systems Medicine, Health and Medical University, Potsdam, Germany; 5 Department of Pharmacy, Center for Drug Research, Ludwig-Maximilians-Universität München, Munich, Germany; 6 Institute for Molecular Medicine, Health and Medical University, Potsdam, Germany; 7 Paul Flechsig Institute for Brain Research, University of Leipzig, Leipzig, Germany; 8 Institute of Human Genetics, University Regensburg, Regensburg, Germany; 9 Metabolomics and Proteomics Core, Helmholtz Zentrum München, German Research Center for Environmental Health, Neuherberg, Germany

**Keywords:** Diabetic retinopathy, Müller glia, mRNA expression profiling, Glucocorticoid receptor signalling, Proteomic analysis

## Abstract

Diabetic retinopathy (DR) is considered a primarily microvascular complication of diabetes. Müller glia cells are at the centre of the retinal neurovascular unit and play a critical role in DR. We therefore investigated Müller cell-specific signalling pathways that are altered in DR to identify novel targets for gene therapy. Using a multi-omics approach on purified Müller cells from diabetic db/db mice, we found the mRNA and protein expression of the glucocorticoid receptor (GR) to be significantly decreased, while its target gene cluster was down-regulated. Further, oPOSSUM TF analysis and ATAC- sequencing identified the GR as a master regulator of Müller cell response to diabetic conditions. Cortisol not only increased GR phosphorylation. It also induced changes in the expression of known GR target genes in retinal explants. Finally, retinal functionality was improved by AAV-mediated overexpression of GR in Müller cells. Our study demonstrates an important role of the glial GR in DR and implies that therapeutic approaches targeting this signalling pathway should be aimed at increasing GR expression rather than the addition of more ligand.

**Graphical Abstract:**

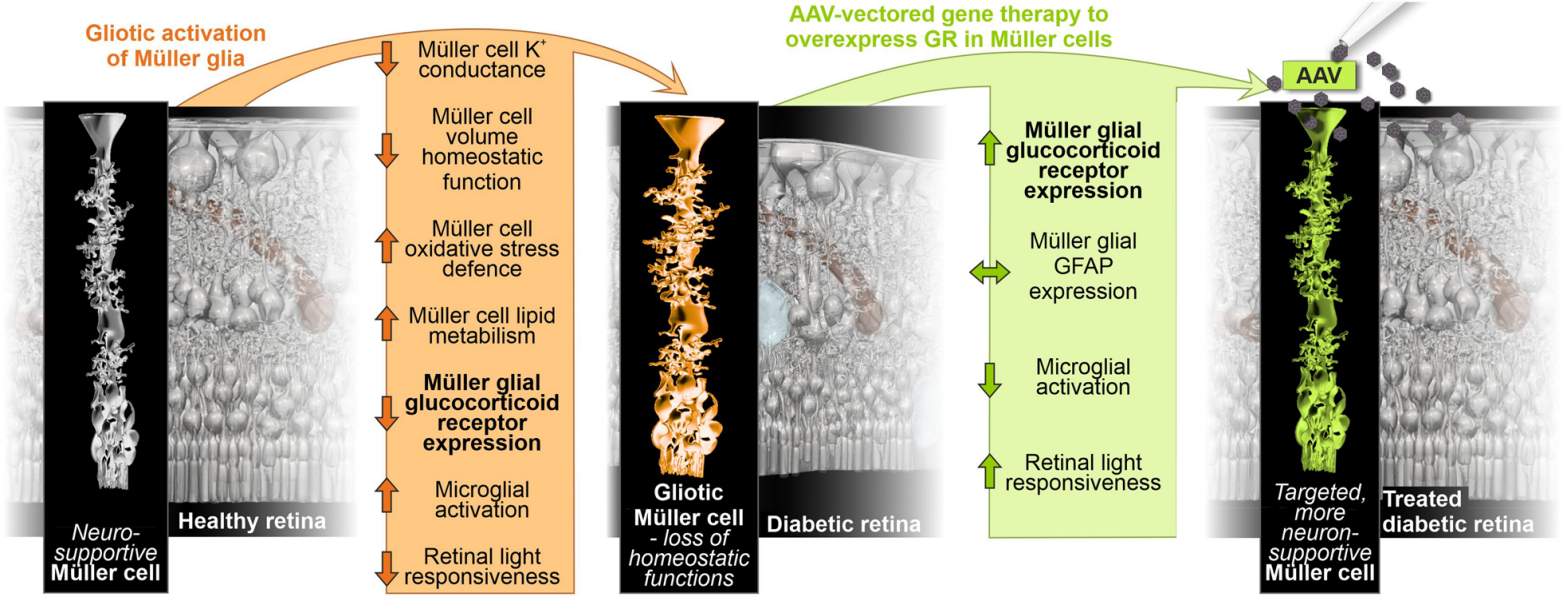

**Supplementary Information:**

The online version contains supplementary material available at 10.1186/s12974-024-03021-x.

## Background

Diabetic retinopathy (DR), the primary cause of blindness in working-age individuals, is a significant complication of diabetes, affecting retinal physiology and vision. Among the 246 million people with diabetes, approximately one third show signs of DR, with a subset developing severe retinopathy or macular oedema [1]. DR manifests initially as non-proliferative, characterised by vascular changes and capillary occlusion, and can advance to a proliferative stage marked by neovascularization, leading to severe visual impairment [2]. A breakdown of the blood-retinal barrier (BRB) leads to intraretinal fluid accumulation and the formation of diabetic macular oedema (DME), which can occur at any stage of DR and is the most common cause of vision loss in DR [2]. Changes in retinal cell types other than vascular cells including Müller cells may lead to oedema formation and neurodegeneration even preceding microvascular remodelling [3]. Among other therapeutic approaches, intravitreal application of glucocorticoids such as triamcinolone acetonide (TA), a selective agonist of the glucocorticoid receptor (GR), are successfully used to reduce cytotoxic oedema in DR. Interestingly, the treatment of Müller cell-ablated mouse models with TA counters effects like photoreceptor degeneration, vascular leakage, and neovascularization, all characteristic of DR. The efficacy of TA in reducing vascular leakage [4], inhibiting the secretion of vascular endothelial growth factor (VEGF; [5], and preventing Müller cell swelling [6] highlights its therapeutic potential in DR.

Although many of the cellular and molecular processes involved in DR have been extensively studied, the interplay between the different retinal cell types and the timing of these interactions are not fully understood [7]. These alterations include an increased flux of glucose through the polyol and hexosamine pathways, accumulation of sorbitol and advanced glycation end products (AGEs), oxidative stress, activation of protein kinase C, chronic unfolded protein response activation, inflammatory responses, dysregulation of the renin-angiotensin system, and activation of the VEGF signalling axis [1, 8, 9].

Müller cells are vital for retinal health, involved in neurotransmitter recycling, metabolic control, and maintaining the BRB [10–13]. Accordingly, selective ablation of Müller cells has been shown to result in early photoreceptor degeneration, vascular abnormalities, breakdown of the BRB and neovascularisation in the retina similar to what is observed in DR [14]. Müller cell dysfunction as observed in multiple preclinical models of diabetes leads to DR-related changes, including decreased glutamate transport and potassium conductance as well as accumulation of iron potentially causing neuronal damage [15–19]. Under diabetic conditions, they also enhance their VEGF and IL-6 secretion, impacting vascular lesions and inflammation in DR, while in contrary IL-6 may also help maintain neuronal function therefore acting as a double-edged sword [7, 20, 21]. Finally, Müller cells contribute to immune responses closely interacting with microglial cells [22, 23].

Central to these changes in Müller cells potentially are signalling mechanisms orchestrated by the GR [24]. The GR, encoded by the Nr3c1 gene, functions as a ligand-dependent transcription factor crucial for glucocorticoid hormone action. Inactive GR, located in the cytoplasm, binds to glucocorticoid ligands, leading to phosphorylation and nuclear translocation for transcriptional regulation [25, 26]. Cortisol, the human endogenous ligand for GR (or corticosterone as the cortisol equivalent in mice), is a key inflammatory regulator, with dysregulated levels observed in diabetic patients, correlating with complications like DR [27–29]. Persistent GR activation can lead to glucocorticoid resistance, diminishing its anti-inflammatory efficacy [26, 30]. In the retina, GR modulation counteracts DR-associated changes like BRB breakdown and neuroinflammation. Studies show that GR activation, via agents like dexamethasone or TA, reduces microglial reactivity, protecting retinal function [31]. Additionally, in a Müller cell-ablated mouse models, selective GR agonists like TA mitigate photoreceptor degeneration and vascular leakage, common in DR [32]. This evidence suggests that the dysregulation of GR in Müller glia is associated with the functional changes typically observed in Müller cells in preclinical models of diabetes, especially given the fact that its highly Müller cell-specific expression has been reported across many species [25].

Here, we report on the role of Müller cells in the context of DR using the db/db mouse with a leptin receptor gene mutation, which develops type 2 diabetes and exhibits DR-like abnormalities [33, 34]. They show elevated insulin levels by 14 days, obesity by 1 month, and hyperglycaemia by 1–2 months of age [35–39]. Features of DR, such as pericyte loss, altered neurovascular coupling, and increased generation of reactive oxygen species, appear at the earliest approximately 1 month after the onset of hyperglycaemia in 3 month old db/db mice [40–42], with increased expression of and VEGF, indicative of vascular remodelling, detected in animals at 5 months of age [43]. More severe signs of DR, such as neurodegeneration and pronounced gliosis, are not observed before 6–7 months of age [44], and BRB breakdown and vascular proliferation are observed even later in 15-month-old db/db mice [45]. We identified pathways that are altered in Müller cells of the db/db retina by analysing changes in glial genome-wide chromatin accessibility in conjunction with the glial transcriptome and proteome during DR progression. This unbiased combinatorial approach identified the GR as a potential master regulator of glial changes in DR. Consequently, we performed AAV-mediated Müller cell-specific GR overexpression and demonstrated that the neuronal deficits observed in the db/db retina can be alleviated by this approach. For this reason, the GR pathway emerges as a master regulator in glial alterations observed in DR highlighting the therapeutic potential of targeting glucocorticoid signalling via gene therapy in Müller cells for DR treatment.

## Results

### Characterisation of the retinal phenotype of db/db mice

**Fig. 1:**
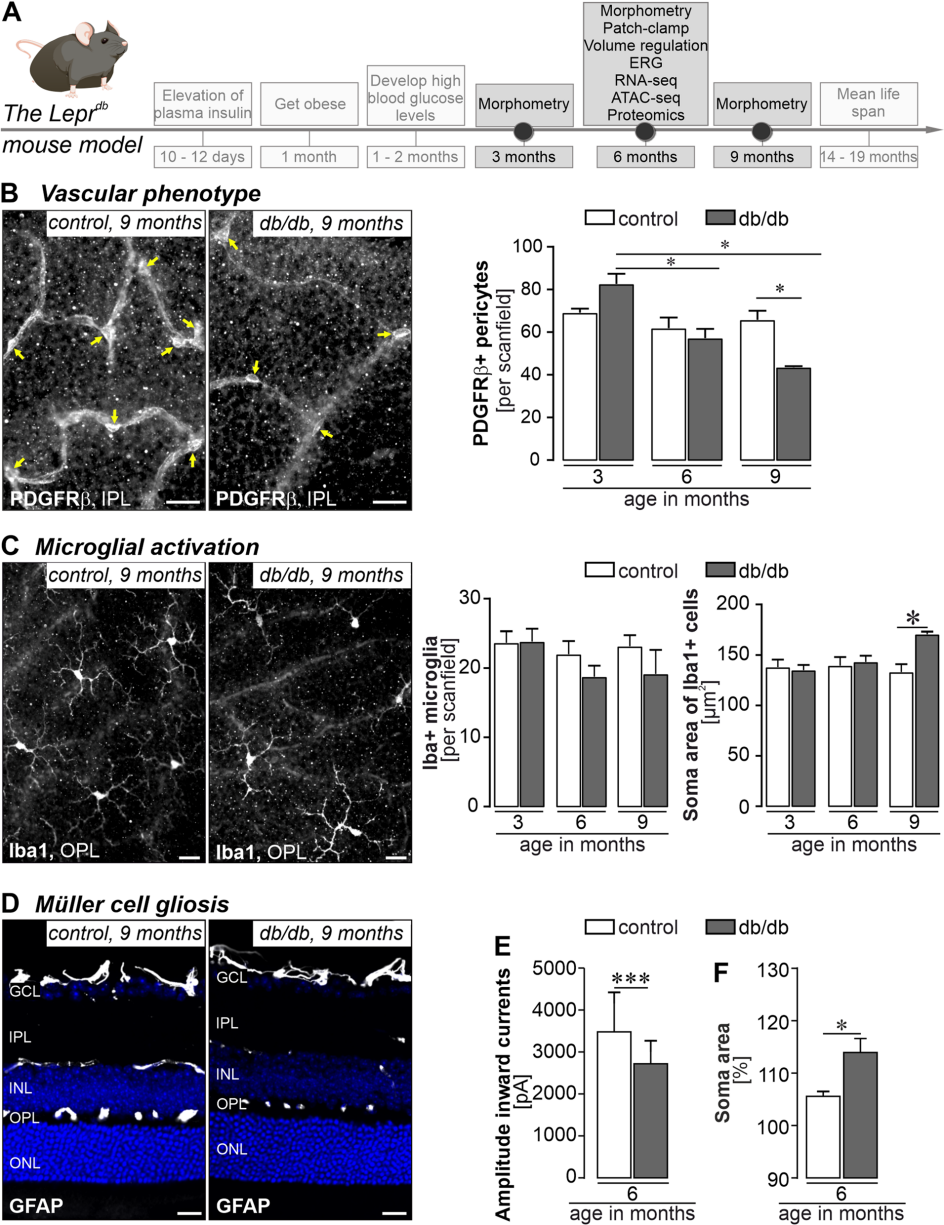
Characterisation of the phenotype of the db/db (Lepr^db^) mice as an animal model for DR in T2D. **A** Timeline of the development of features of T2D and DR in the db/db mouse model. Time points of data collection are highlighted as dots on the time line and respective readouts are listed in the boxes above. **B** *Left*, representative micrographs of PDGFRβ staining to delineate pericytes (yellow arrows) in retinal flatmount preparations of diabetic and control mice 9 months of age. Scale bar, 20 µm. *Right*, PDGFRβ-positive cells were counted per scan field (410 µm × 298 µm) of both genotypes. Bars represent mean ± SEM and comprise data from the following number of biological replicates: 3 months of age, *n* = 4 mice per genotype; 6 months of age, *n* = 6 mice per genotype; 9 months of age, *n* = 3 mice per genotype.. Unpaired *t*-test: *p<0.05. **C** *Left*, representative micrograph of Iba1 stainings of retinal flat mounts from 6-month-old diabetic and control mice. Scale bar, 20 µm. *Right*, Iba1-positive cells were quantified per scan field (410 µm × 298 µm). Z-scans through the whole thickness of the retina were performed and cells across all retinal layers were counted. In addition, the soma area of each microglia in a scan field was measured as an indicator of beginning microglial activation. Bars represent mean ± SEM and comprise data from the following number of biological replicates: 3 months of age, *n* = 4 mice per genotype; 6 months of age, *n *= 6 mice per genotype; 9 months of age, *n* = 3 mice per genotype. Unpaired *t*-test: *p<0.05. **D** GFAP was only present in astrocytes residing in the nerve fibre layer, but not in Müller cells—neither in healthy controls nor in retinae from db/db mice 6-months of age. **E** Patch clamp recordings of isolated Müller cells from 6-month-old db/db mice demonstrated a significant reduction of amplitudes known to be primarily mediated by Kir4.1 potassium channels. Bars represent mean ± SD (standard deviation) and comprise data from ~ 40 cells collected from 4 animals per genotype. Mann–Whitney-test: ***p<0.01. **F** The ability of Müller cells to compensate for hypoosmotic stress was tested in the retina of 6-month-old db/db mice. Vital retinal sections were exposed to hypoosmolar stress (60% osmolarity) for 4 minutes. Changes in the Müller cell soma area, visualised by labelling with Mitotracker Orange, were measured as an indication of cell swelling. Bars represent mean ± SEM and comprise data from ~15 cells collected from 2 animals per genotype. Mann–Whitney-test: *p < 0.05.

To validate data from the literature and further characterise the retinal phenotype of the db/db mouse strain, morphometric analysis was performed on eyes from db/db and control animals at 3, 6, and 9 months of age which reflects 1, 4 and 7 months of diabetes duration (Fig. 1A). An important hallmark of diabetes is early changes in the integrity of the microvascular system, which are also observed in the progression of DR [46]. Using trypsin-digested retinal flatmounts stained with haematoxylin and eosin (H&E), we analysed the changes in the microvascular system of our db/db breed. Flat mounts from 3- and 6-month-old db/db animals and controls were used to quantify acellular capillaries (lagging pericytes and endothelial cells), but no significant differences were found at all ages analysed (Additional file 1: Fig. S1). A significant increase in the endothelial cell/pericyte ratio was calculated for 6-month-old diabetic animals compared to controls. In our db/db strain, only a mild decrease in pericyte number was observed in 6-month-old animals that became more pronounced in 9-month-old db/db mice (Fig. 1B). This age-dependent loss was not observed in wild-type controls at any age.

We next examined whether microglia activation can be detected as reported by others [37]. We found no significant difference in the number of Iba1-positive microglia/macrophages in the retinae of db/db and control animals (Fig. 1C). To enable cell type-specific molecular profiling, we then purified microglia, Müller cells, vascular cells, and retinal neurons by magnetic-activated cell sorting (MACS) from 6-month-old mice of both genotypes and subjected the samples to RNA sequencing (RNA-seq, 25–45 million reads per sample) and MS/MS mass spectrometry analysis. Purification of cell types was validated by plotting the expression of known marker genes (Additional file 1: Fig. S2A–D). Principal component analysis (PCA) revealed that samples of the four cell types analysed clustered separately based on their transcriptome (Additional file 1: Fig. S2B), suggesting that cell identity dominates over diabetes-related changes in expression. Importantly, we found a significant upregulation of the microglia/macrophage marker *Itgam* (alias C11b) at transcript and protein levels in purified microglia/macrophages from db/db mice at 6 months of age (Additional file 1: Fig. S2A, B), as well as a consistent, although not yet always significant, upregulation of transcripts of additional markers of microglial activation, including *Aif1*, *Il1b*, *Trem2*, and *C1qa *(Additional file 1: Fig. S2E). The area of the microglial somata was significantly larger only in the 9-month-old diabetic animals than those in age-matched control mice (Fig. 1C), confirming microglial activation at the cell morphological level at later stages than detected at the molecular level.

GFAP upregulation was not yet detectable by immunostaining in Müller cells of in 6-month-old db/db mice, while astrocytes in the nerve fibre layer were GFAP immunopositive (Fig. 1D). However, by analysing purified Müller cells, we demonstrate a moderate but significant upregulation of Gfap  using RNA-seq and proteomic data (Additional file 1: Fig. S2F). The expression of Müller cell gliosis genes known to be dysregulated in retinal pathologies such as retinitis pigmentosa, but also in DR, such as *Stat3*, *S100a1* or *CD44*, were plotted to further validate the gliotic activation in our db/db mice at the molecular level [47–49]. Here we found the expected pattern of gene up- or down-regulation, even though not all changes reached the significance level. Importantly, we also monitored functional changes typically observed in gliotic Müller cells. First, patch clamp recordings from acutely isolated Müller cells of 6-month-old animals showed a significant decrease in potassium channel-mediated inward currents in db/db mice indicating their gliotic activation (Fig. 1E). Second, and consistent with the results from patch clamp recordings, the ability of Müller cells in these diabetic animals to regulate volume was also severely impaired, resulting in increased soma size upon exposure to hypoosmotic stress (Fig. 1F). Thus, we confirm early functional changes in Müller cells indicative of an onset of Müller cell gliosis at about the same age when the first signs of vascular changes appear.

**Fig. 2:**
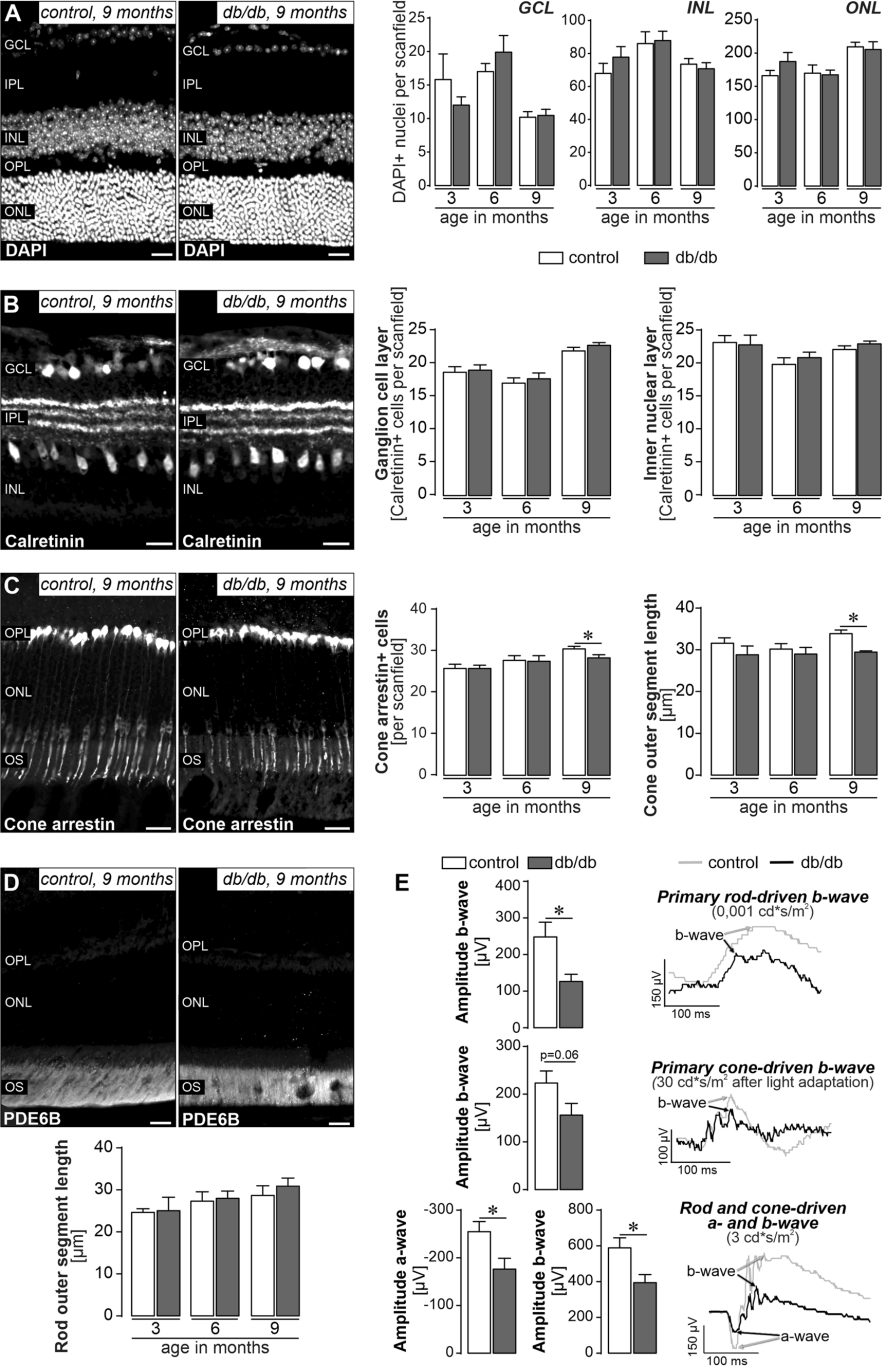
In-depth analysis of morphological and functional changes of retinal neurons in db/db mice. **A** *Left*, representative micrograph of a DAPI-staining of the retina of 9-month-old diabetic and control mice. *Right*, quantification of DAPI-positive nuclei in the three nuclear layers of the retina does not reveal major cell loss even in 9-month-old db/db mice. Scan field: 68 µm × 200 µm. **B** *Left*, calretinin immunolabeling delineates ganglion and displaced amacrine cells in the ganglion cell layer (GCL) and amacrine cells in the inner nuclear layer (INL). Representative micrographs of retinae from 9-month-old diabetic and control mice are shown. Right, the number of calretinin-positive cells per scan field of the GCL and INL is plotted. Scan field: 200 µm × 200 µm. **C** Left, cone photoreceptors including their outer segments are visualized by a cone-arrestin staining for which representative results are presented from 9-month-old animals. Scale bar, 20 µm. Right, the number of cones and the length of their outer segments (OS) was assessed in retinae from both genotypes. Unpaired *t*-test: *p < 0.05. **D** *Top*, representative micrograph of PDE6B immunoreactivity in rod OS in retinal sections from 9-month-old diabetic and control mice. *Bottom*, the rod OS length of retinae from db/db and control animals was measured. **E** Electroretinogram (ERG) recordings were performed on 6-month-old animals. Scotopic rod-specific b-wave, photopic cone-specific b-wave and mixed rod-cone–specific a- and b-wave were measured and quantified at 0,001 cd/ms, 30 cd/ms and 3 cd/ms, respectively. Right, representative ERG traces. Bars represent mean values ± SEM. *N* = 10 control animals and *n* = 11 db/db mice analysed. Unpaired *t*-test: *p < 0.05. **A**–**D** Scale bars, 20 µm. Bars represent mean ± SEM and data from 4 animals per age and genotype are shown, except for 9-month-old db/db mice where only 3 biological replicates were analysed. *IPL* inner plexiform layer, *OPL* outer plexiform layer, *ONL* outer nuclear layer, *OS*, outer segments

We then addressed the question whether the microvascular and glial alterations result in neurodegeneration. We quantified cell numbers in the ganglion cell layer (GCL), inner nuclear layer (INL), and outer nuclear layer (ONL) (Fig. 2A). We found no significant change in cell numbers in our db/db breed compared to control animals at any of the ages studied (Fig. 2A). To investigate neurodegeneration at the cell type level, we assessed the number of calretinin-positive cells representing ganglion cells and displaced amacrine cells in the GCL and amacrine cells in the INL [50, 51], but again did not find significant differences between genotypes (Fig. 2B). Finally, we examined rod and cone photoreceptors known to be particularly susceptible to degenerative processes in multifactorial diseases such as DR [52]. The total number of cones per scan field and the length of their outer segments were slightly reduced in retinae from diabetic animals at 9 months of age (Fig. 2C). We also measured the length of rod (outer and inner) segments based on PDE6B immunolabeling and found no difference between genotypes at any age investigated (Fig. 2D).

Next, we determined diabetes-associated changes on the functional integrity of the retinal tissue. Electroretinogram (ERG) recordings were performed in 6-months-old animals—a time point at which no major anatomical changes could yet be observed, but initial changes in the microvascular system (Fig. 1B), microglial activation (Additional file 1: Fig. S2E) and Müller cell expression profiles (Additional file 1: Fig. S2F) and function (Fig. 1D) were detected. The rod (scotopic)-driven b-wave amplitude was significantly smaller in diabetic mice than in controls (Fig. 2E). The cone b-wave amplitude (photopic) was also reduced in diabetic mice, however, the effect was not statistically significant (*p* = 0.06). The mixed responses of cones and rods under mesopic light conditions were significantly reduced in diabetic mice for both a- and b-waves (Fig. 2E).

Overall, our data suggest that the progression of neurodegenerative processes as a hallmark of DR in the db/db mouse strain is slow, given that significant neurodegeneration occurs not before 9 months of age.

### Early changes in the glial transcriptome and proteome of diabetic retinae suggest metabolic dysregulation and altered growth factor signalling

The primary goal of this study was to define the gene expression signature of Müller cells in early pre-DR. In light of their central role as a component of the retinal neuro-vascular unit primarily affected in DR, we focussed the in-depth cell type-specific transcriptome and proteome analysis on Müller cells isolated from 6-month-old animals. This is the time point 4 months after the onset of T2D when, according to our phenotypic validation, Müller cell homeostasis functions are already significantly diminished. However, even though neuronal function is reduced as shown by ERG recordings, significant neuronal loss and vascular changes have not yet occurred and could be rescued by a treatment targeting the early Müller cell dysfunction.

**Fig. 3:**
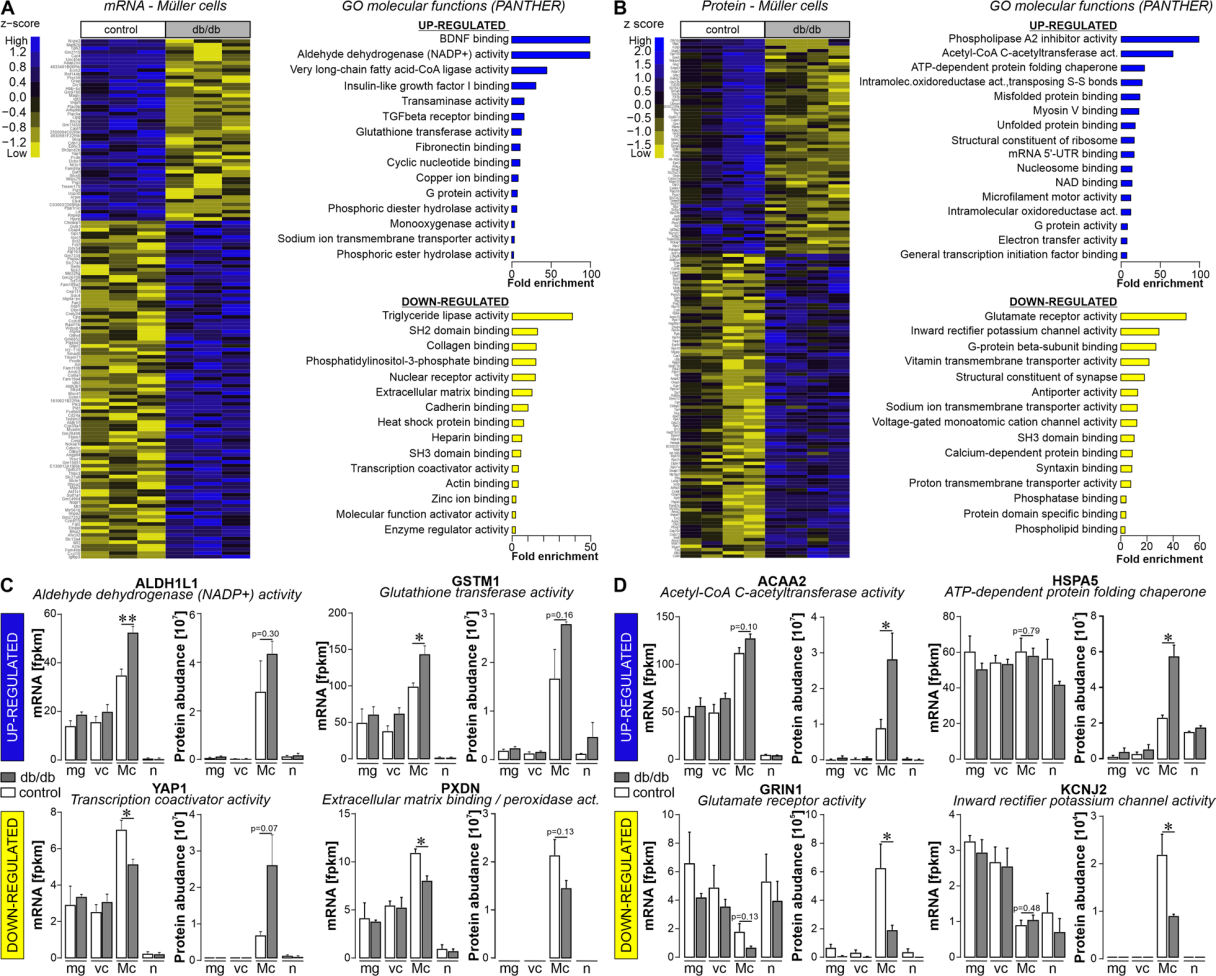
Expression landscapes at transcript and protein level of purified retinal Müller cells isolated from diabetic mice 6 months of age hint towards metabolic changes and altered growth factor signalling. **A** *Left*, Differentially expressed genes of Müller cells isolated from 6-month-old db/db mice compared to controls (*p* < 0.05; at least twofold difference), which additionally showed Müller cell specificity (*p* < 0.05 compared to expression in neurons; at least twofold difference is plotted in the heatmap). The heatmap includes three biological replicates per genotype to illustrate the degree of heterogeneity in gene expression within each genotype. *Right*, GO molecular functions (based on fold enrichment, at least twofold) of significantly up- or down-regulated genes in Müller cells from 6-month-old db/db mice as determined by pathway enrichment analysis using PANTHER. **B** *Left*, filtering (*p* < 0.05; at least twofold difference) was performed to identify proteins differentially regulated in Müller cells from 6-month-old diabetic mice. Data from 4 biological replicates per genotype are plotted. *Right*, molecular functions driven by proteins significantly down- or up-regulated in Müller cells from 6-month-old db/db mice determined by pathway enrichment analysis using PANTHER. **C** Transcript and protein expression of select candidate genes central to the pathways as identified from differentially expressed Müller cell-specific transcripts shown in **A** are plotted across all cell types to illustrate Müller cell-specificity of the diabetes associated gene expression. Bars represent the mean ± SEM of three biological replicates per genotype for RNA-seq data and 4 biological replicates per genotype for protein abundances, respectively. Unpaired *t*-test: *p < 0.05, **p < 0.01. **D** Transcript and protein expression of select candidate genes central to the pathways as identified on the proteome profiles shown in **B** are plotted across all cell types to illustrate Müller cell-specificity of the diabetes associated gene expression change. Bars represent the mean ± SEM of three biological replicates per genotype for RNA-seq data and 4 biological replicates for protein expression. Unpaired *t*-test: *p < 0.05. **C**, **D** mg, microglia; vc, vascular cells; Mc, Müller cells; n, neurons

A PANTHER (released 20221013) overrepresentation test was used to perform pathway enrichment analysis on GO molecular functions of Müller cell-specific genes (i.e. twofold versus neuronal expression levels, *p* < 0.05) that were significantly upregulated (117 genes) or downregulated (68 genes) in diabetic retina (Additional file 1: Table S1). Genes related to aldehyde dehydrogenase (NAD+/NADP+), and glutathione transferase activity, in addition to several growth factor pathways, including brain-derived growth factor (BDNF), transforming growth factorβ (TGFβ) and insulin-like growth factor 1 (IGF1) as well as G-protein-mediated signalling, were upregulated in Müller cells from diabetic mice (Fig. 3A, Additional file 1: Table S1). Pathways associated with downregulated genes included those involved with lipid metabolism (triglyceride ligase activity), extracellular matrix and cell–cell interactions (extracellular matrix binding, collagen binding, heparin binding, cadherin binding, actin binding), intracellular signalling (e.g. src homology (SH) regions 2 and 3 domain binding, phosphatidylinositol-3-kinase binding), and gene expression regulation (Fig. 3A, Additional file 1: Table S1).

We also subjected retinal cell populations purified by magnetic-activated cell sorting (MACS) to unbiased label-free liquid chromatography-mass spectrometry proteome profiling. To maximise comparability, the same neuronal, microglial, vascular and Müller cell marker genes were selected as previously used to validate cell enrichment in the RNA-seq dataset. This convincingly confirmed the successful separation of the different retinal cell populations (Additional file 1: Fig. S2C). PCA based on the cell type-specific proteomes showed that cell populations formed four clearly separated clusters (Additional file 1: Fig. S2D). The proteins which were significantly up (92 proteins)- or down (66 proteins)-regulated in Müller cells of 6-month-old diabetic mice were identified (Fig. 3B, Additional file 1: Table S2). Analysis of the enrichment of down-regulated proteins by PANTHER shows down-regulation of adenosine triphosphatase (ATP) and transporter activity (Fig. 3B). In contrast, pathways related to RNA, protein binding, ligase, and transferase activity were associated with proteins upregulated in Müller cells from diabetic mice (Fig. 3B).

Since we generated the transcriptomic and proteomic data in a highly comparable manner with respect to the cell isolation protocols and the mouse strain used, we asked to what extent changes in gene expression at the transcriptional level are actually reflected in the proteomes of the cells. A total of 883 Müller cell-specific genes (56.4%) present in both data sets showed a congruent expression profile at the mRNA and protein level (Additional file 1: Fig. S3A). Focussing on the genes that were differentially expressed in Müller cells of the diabetic retina resulted in similar proportion of genes (57.3%) that were found to have a consistent regulation at the mRNA and protein level (43 out of 75 differentially expressed genes (DEGs, Additional file 1: Fig. S3B). The partial mRNA/protein discrepancy for some DEGs is also reflected in the expression profiles of selected candidates that were central to pathways discussed above (Fig. 3C, D). While upregulation of aldehyde dehydrogenase 1 family member L1 (*Aldh1l1*) or glutathione S-transferase mu 1 (*Gstm1*) was specific to Müller cells and consistent in both transcript and protein expression, significant downregulation of Yes1-associated transcriptional regulator (*Yap1*) at the RNA level was not reflected in protein expression, but YAP1 protein levels were higher in db/db Müller cells compared to wild-type cells. Finally, the down-regulation of peroxidasin (*Pxdn*), a heme-containing peroxidase that is secreted into the extracellular matrix, was consistent again for transcript and protein expression. Focussing on the DEGs driving the pathway analysis shown in Fig. 3B, we found a similar discrepancy between transcript and protein expression. Consistent expression was found for mitochondrial acyl-CoA hydrolase (*Acaa2*), an enzyme that catalyses the final step of mitochondrial fatty acid beta-oxidation, which was significantly and specifically upregulated in Müller cells (Fig. 3D). Also for glutamate ionotropic receptor NMDA Type Subunit 1 (*Grin1*), a significantly down-regulated protein in Müller cells, the regulation of the transcript matches the protein. While *Grin1* transcripts were rather equally detected in all cell populations, the protein was almost exclusively present in Müller cells. In contrast, transcripts of heat shock 70 kDa protein 5 (glucose-regulated protein, 78 kDa;* Hspa5*) and inward rectifier potassium channel 2 (alias Kir2.1, *Kcnj2*) were not differentially expressed or even specific to Müller cells, while the respective protein was significantly up- or down-regulated and specific to the Müller cell population when compared to microglia, vascular cells or neurons (Fig. 3D). The cytoskeletal-associated protein 4 (*Ckap4*), a novel RNA-binding protein [53], is the only Müller cell-specific gene we found to be consistently and significantly dysregulated at both mRNA and protein level (Additional file 1: Fig. S3B, C, Tables S1, S2).

### The GR was identified as factor potentially driving changes in the Müller cell gene expression pattern in the diabetic retina

**Fig. 4:**
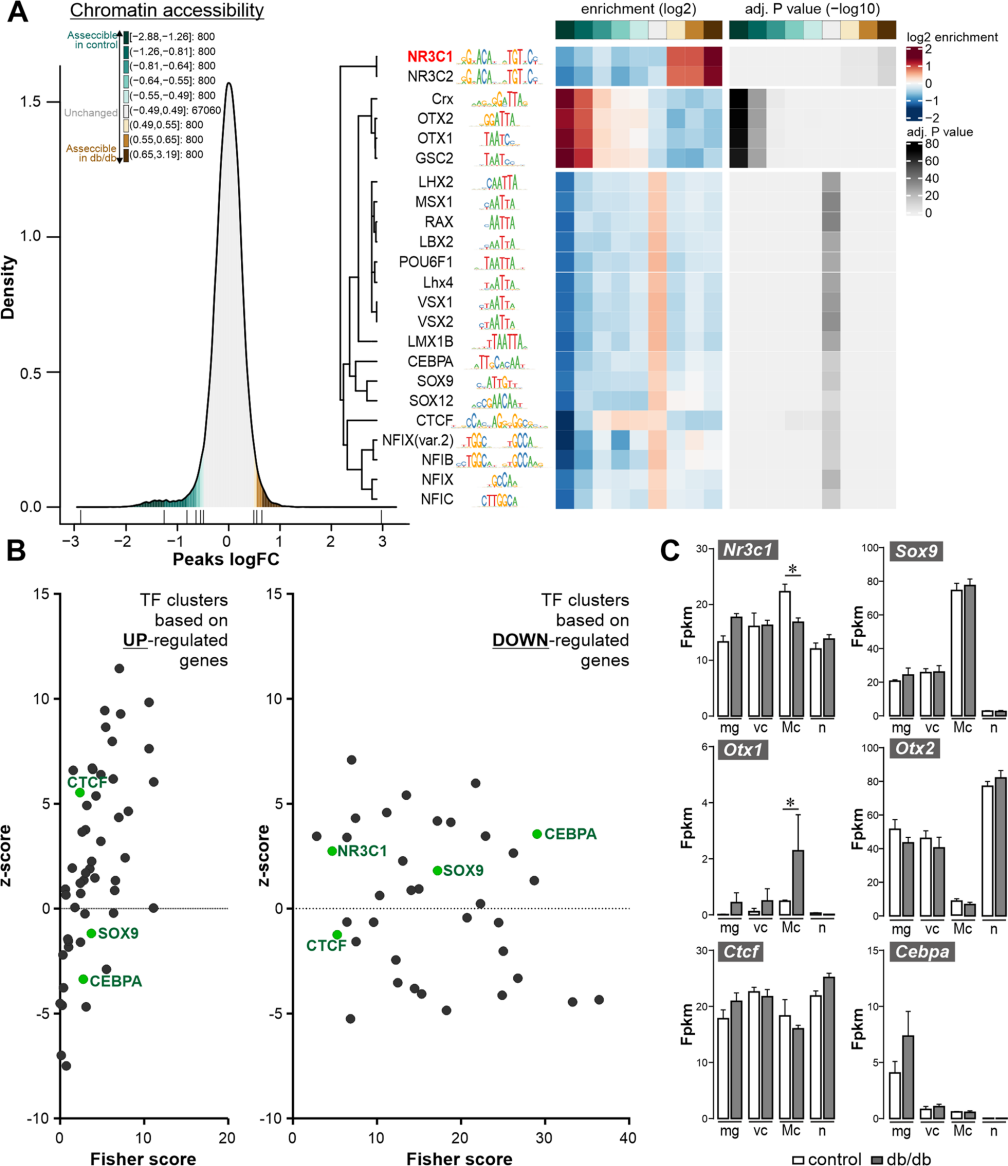
Identification of the GR (gene ID: *Nr3c1*) as a putative key regulator of glial transcriptomic changes in diabetic mice on basis of chromatin accessibility and RNA expression profiles. **A** Chromatin accessibility was assessed by ATAC-seq on purified Müller cells from 6-month-old mice of both genotypes (2 biological replicates per genotype). Data were subjected to transcription factor binding motif analysis. *Left*, Density plot showing the distribution and colour code of bins comprising an equal number of peaks corresponding to fold changes in chromatin accessibility between db/db and control mice. Numbers represent the log2 fold change range (db/db vs control) and the corresponding number of peaks per bin. *Right,* Cluster analysis and heat map of transcription factor (TF) binding motifs significantly enriched when comparing chromatin accessibility of Müller cells from db/db mice with their counterparts from wild-type animals. Bins in shades of green indicate decreased accessibility, while shades of brown indicate increased accessibility. Motifs enriched in the grey bin are those with unchanged chromatin density between genotypes. **B** TF clusters possibly involved in regulatory changes associated with DR progression as identified by oPOSSUM-3 analysis. Genes/transcripts identified by RNA seq to be significantly up- or down-regulated in Müller cells of 6-months-old diabetic mice were submitted to oPOSSUM-3 analysis [56]. Only TFs that were expressed at transcript level in Müller cells are included. TFs identified in both ATAC-seq and oPPOSSUM-3 analysis are highlighted in green. **C** Expression levels of TFs highlighted in green in **B** and of *Otx1* and *Otx2* as two examples of the TFs that were prominent in **A** by a high-level reduction in the accessibility of their DNA-binding domains. Data are from the RNA-seq dataset performed on MACS-sorted cells (see Fig. 3). Bars represent mean ± SEM from 3 biological replicates per genotypes (one dot per replicate). Unpaired *t*-test: *p < 0.05. *Mg* microglia, *vc* vascular cells, *Mc* Müller cells, *n* neurons

To identify key regulators of Müller glial changes in DR progression, we investigated changes in DNA accessibility by performing ATAC-seq on purified Müller cells from 6-month-old mice and subjected these data to transcription factor (TF) binding motif analysis. Motifs for the GR (gene symbol: *Nr3c1*) stand out as being more accessible in Müller cells isolated from diabetic retinae compared to controls (Fig. 4A). In contrast, motifs for TFs that are known to determine and maintain photoreceptor identity (*Crx, Otx1, Otx2*) were less open in cells isolated from db/db retinae [54]. Since some contamination of our Müller cell population by photoreceptors cannot be avoided [55], this latter finding may also reflect early changes in photoreceptor gene expression signatures long before functional (Fig. 2E) or even morphological (Fig. 2C) findings are observed. Finally, motifs for two members of the NFI family—*Nfix (var. 2)* and *Nfib,* as well as *Ctcf* were enriched in regions which were accessible in both control and diabetic cells (Fig. 4A).

Next, the search for key regulators of Müller glial changes in DR progression was approached from the mRNA perspective. An oPOSSUM-3 TF binding site cluster analysis [57] based on differentially regulated genes in Müller cells from 6-month-old diabetic mice as identified by the RNA-seq experiment identified multiple gene clusters that are upregulated in Müller cells, e.g. *Sp1*, *Ebf1*, *Egr1*, *Zfx*, *Sox9* and *Foxd1* (Fig. 4B, Additional file 1: Table S1), and gene clusters related to down-regulated transcripts including *Sox9*, *Rora*, *Foxf2*, *Srf* and *Nr3c1* (Fig. 4B, Additional file 1: Table S1).

Of note, several TFs, including *Nr3c1, Sox9, Ctcf,* and *Cebpa* (highlighted in green, Fig. 4), were identified via oPOSSUM and ATAC-seq approaches, making them potentially interesting candidates as master regulators of glial gene expression signatures in DR. *Nr3c1* (GR) transcripts, while present across all cell types, were most abundant in Müller cells, and were significantly reduced in 6-month-old db/db mice as compared to age-matched controls (Fig. 4C). *Sox9* transcripts were even stronger enriched in Müller cells than that of the GR, but no change of its expression level was obvious between genotypes investigated. *Ctcf*, in fact a putative target gene of *Nr3c1*, was also uniformly expressed in all retinal cell types, with the least of its transcripts detected in Müller cells. In Müller cells from diabetic mice, *Ctcf* expression was slightly lower than in controls, although this trend did not reach significance. *Cebpa* was found to be expressed specifically in microglia with a trend of upregulation in cells isolated from 6 months old mice. Finally, two out of the four TFs, for which highly significant closure of DNA binding motifs was identified (Fig. 4B), were also added. *Otx1* transcripts were expressed at low levels in Müller cells (Fig. 4C). Interestingly, significantly more transcripts were detected in cells isolated from 6-month-old diabetic mice. In contrast, Otx2, which together with the well-established photoreceptor-specific TF *Crx*, is a known driver of photoreceptor differentiation and controls the expression of rhodopsin in rods [58, 59], was significantly enriched in the neuronal population (Fig. 4C). However, no significant effect of the diabetic condition on its expression level was detected in any of the cell types, including the rod-rich neuronal population [55].

Ultimately, the GR stands out from all TFs mentioned because (i) its DNA binding motifs show altered accessibility with progression of DR, (ii) it could also be identified as a potential master regulator via our Müller cell-specific RNA-seq dataset, as target genes of the GR were enriched amongst the differentially expressed Müller cell-specific genes, and (iii) the GR is expressed at comparatively high levels in Müller cells.

### In depth characterisation of the GR as potential master regulator of Müller cell gene expression

**Fig. 5:**
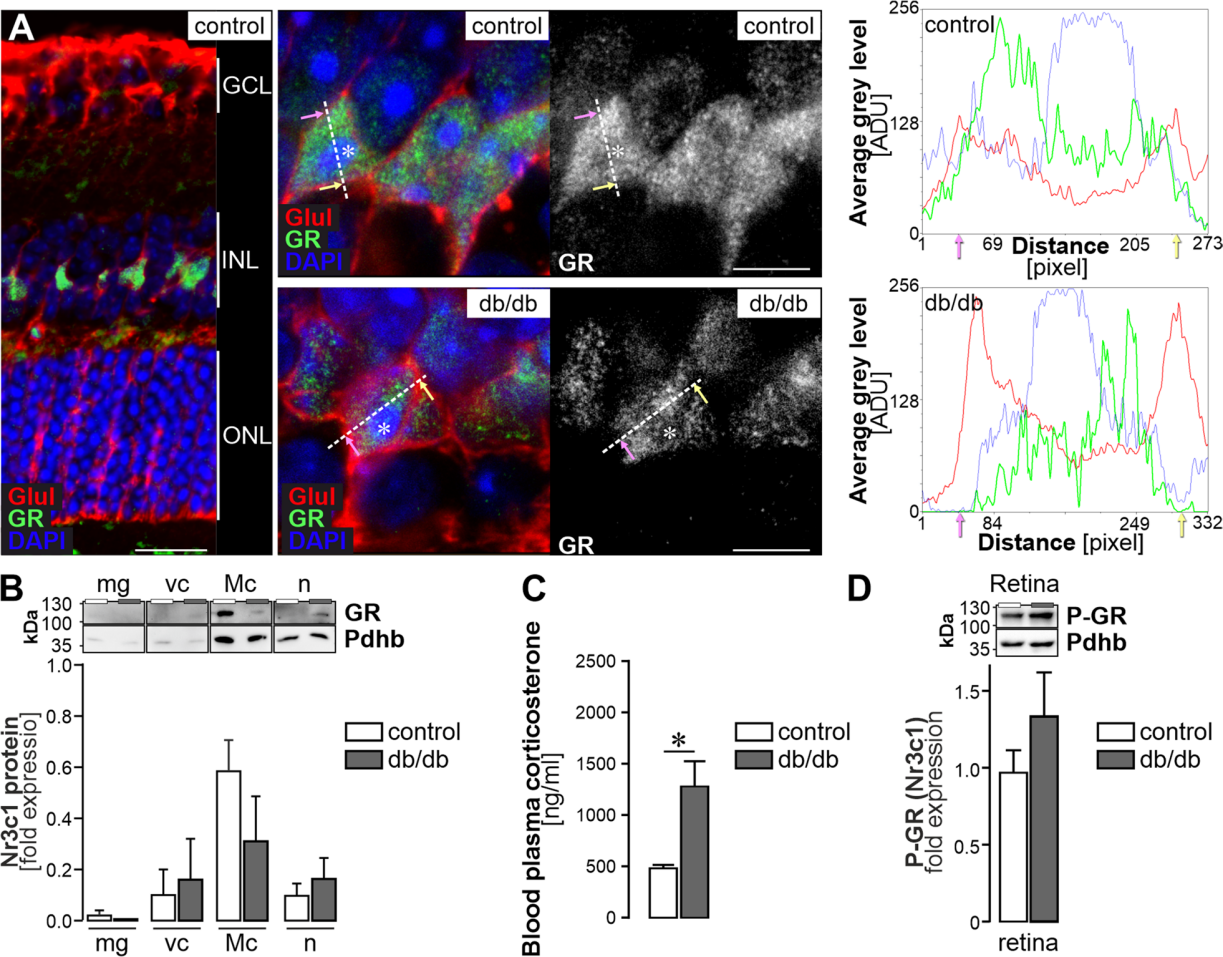
The GR is specifically expressed in Müller cells and modulates glial gene expression. **A** *Left*, Confocal image of a GR labelling in retinal section of a 6-month-old mouse. Scale bar, 20 µm. *Middle*, STED images of a GR staining in the inner nuclear layer (INL). Müller cells were co-stained for glutamine synthetase (GLUL). Scale bars, 5 µm. *Right*, Line plots of mean grey values for each channel (red—GLUL as Müller cell marker residing in the cytoplasm; green—GR; blue—DAPI highlighting the DNA condensed in the nucleus). The dashed line indicates the plane of the line scan in the respective micrographs. Pink and yellow arrows indicate the orientation of data plotted in the histograms in relation to the actual line set in the micrograph. Asterisks highlight nucleoli. GCL, ganglion cell layer; INL, inner nuclear layer; ONL, outer nuclear layer. **B** Western blot to detect GR in purified retinal cell types isolated from 6-month-old mice. As the protein yield per cell population isolated per two pooled retinae is very low, the whole protein extract per cell pellet was loaded. To enable quantification of the GR band, the signals were normalised to the house keeper PDHB as done in previous studies implementing MACS-purified retinal cell types [60]. Bars represent mean ± SEM (*n* = 6 biological replicates per genotype). **C** The corticosterone level was measured in the blood plasma of diabetic and control animals at an age of 6 months via ELISA. Results of 4 control and 5 db/db mice are plotted as mean ± SEM. Unpaired *t*-test: *p < 0.05. **D** Western blot of phosphorylated GR performed on whole retinal extracts from animals 6 months of age. Bars represent mean ± SEM (*n* = 4 animals per genotype)

Given the results from ATAC-seq and oPOSSUM analysis, we first aimed to confirm that the GR is expressed in Müller cells and is differentially expressed in the diabetic retina. GR-immunoreactivity was clearly localised to Müller cell somata in the INL and, the staining intensity seems weaker in sections from 6-month-old diabetic mice (Fig. 5A). Super resolution imaging by STED shows little overlap of GR signals in Müller cell somata with signals from cytoplasmic glutamine synthetase, but a broad overlap with nuclei visualised by the DAPI staining. Interestingly, GR does not seem to be present in nucleoli (Fig. 5A), but locates primarily in the less condensed euchromatin of the nucleus consistent with the function of a transcription factor. Western blots performed on purified retinal cell populations from control and diabetic mice at 6-months of age confirmed the findings from the immunostainings that the GR is also down-regulated at protein level in Müller cells of the diabetic retina (Fig. 5B, Additional file 1: Fig. S4A). Interestingly, we detected a significantly higher plasma corticosterone level in 6-month-old db/db animals as compared to controls (Fig. 5C). Next, we asked whether the remaining GR could be more active due to a surplus of available ligand. To this end, we measured GR-phosphorylation in whole retina protein extracts from 6-month-old animals and found a trend of increased activation in db/db mice (Fig. 5D, Additional file 1: Fig. S4 B).

**Fig. 6:**
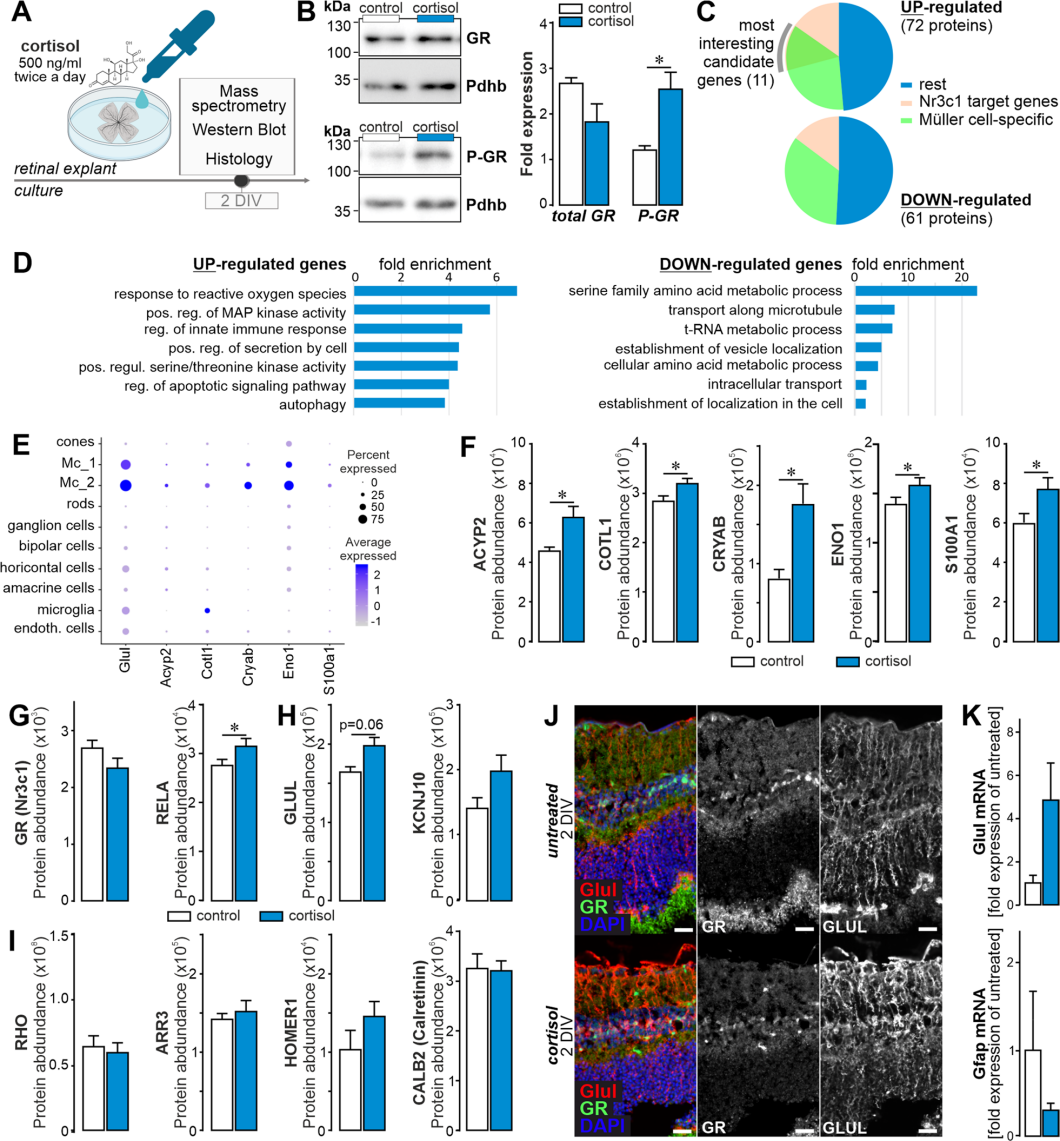
Cortisol treatment of wild-type mouse retinal explants reinforces GR signalling and enhances glial homeostatic gene expression. **A** Experimental scheme of retinal explant cultures with persistent GR activation. **B** *Left,* Representative Western blots performed on whole retinal explant protein extracts when cortisol (500 ng/ml) was added twice daily. *Right*, Quantification of the total amount of GR and P-GR protein levels at 2 days of cortisol treatment. PDHB served as housekeeper to which the GR and P-GR were normalised to. Results of *n*=3 explants per treatment group are plotted as mean ± SEM. Unpaired *t*-test: *p<0.05. **C** Retinae from 6 control mice were cut into half. One half remained untreated and was kept under standard culture conditions. The other half was treated with cortisol (500 ng/ml, supplemented twice a day). Mass spectrometric profiling of explants after cortisol treatment over 48 h (*n*=6 biological replicates per treatment group) revealed 133 differentially expressed proteins. Using JASPER, ENCODE and CHEA databases, we identified those proteins whose genes are putative targets of GR and checked if those were then Müller cell-specific basing on our own RNA-seq data of purified retinal cell types. Eleven overlapping proteins were found amongst the up-regulated candidates, but none amongst the down-regulated upon cortisol treatment. **D** Pathway enrichment analysis of differentially expressed proteins from (**D**) using STRING. **E** Reanalysis of single cell RNA-seq data from Macosko et al. [64] confirmed Müller cell-specific expression of many of the 11 GR target genes found to be up-regulated upon cortisol treatment. **F** Protein quantification via mass spectrometry (*n* = 6 biological replicates per treatment group) of putative Müller cell-specific GR target genes as identified by filtering of data in (**D**). Unpaired *t*-test: *p<0.05. **G–I** Protein expression levels of select candidates are plotted on basis of quantitative mass spectrometric data collected from cortisol-treated retinal explants (*n* = 6 biological replicates per treatment group). Besides Nr3c1, its interaction partner RELA was chosen (**H**), and additionally Müller cell (**I**) and neuronal marker genes (**J**). GLUL, glutamine synthetase; KCNJ10, Kir4.1; RHO, rhodopsin; ARR3, cone arrestin; HOMER1, homer scaffold protein 1; CALB2; calretinin. **J** Sections from retinal explants were stained for the Müller cell marker glutamine synthetase (GLUL) and GR. **K** Quantitative real-time PCR (qPCR) of Müller cells purified form retinal explant cultures at 2 DIV was performed to determine mRNA levels of Glul and Gfap. Bars represent mean ± SEM (*n* = 3 for each condition). Scale bars, 20 µm.

To determine whether increased exposure to GR ligands affects the expression profile of Müller cells, retinal explants of wild-type mice were kept in culture and cortisol was supplemented (Fig. 6A), which led to higher levels of GR activation indicated by its enhanced phosphorylation (Fig. 6B, Additional file 1: Fig. S4C). For unbiased analysis of the putative GR gene regulatory network, we performed mass spectrometric protein profiling on explant cultures after 2 days of cortisol treatment. 72 proteins were found to be significantly upregulated and 61 were down-regulated (Fig. 6C). Pathway enrichment analysis revealed genes involved in ion and vesicular transport were down, while oxidative stress defence and regulation of inflammatory pathways were up-regulated upon cortisol treatment (Fig. 6D). Eleven of the upregulated genes were potential direct targets of GR as reported by the three databases JASPER, ENCODE, and CHEA [61–63] and matched with gene expression changes upon cortisol treatment. Cross validating the Müller cell-specificity of these 11 candidates, we checked published scRNA-seq resource data from mouse retina [64] (Fig. 6E).

Five candidate genes were predominantly expressed in Müller cells and were significantly up-regulated upon cortisol treatment (Fig. 6F). This supports the idea that modulation of GR signalling can directly affect Müller cell-specific genes. Finally, we analysed GR protein and interacting RELA expression in the cortisol-treated retinal explants. GR expression was slightly reduced (confirming the assumption of an autoregulatory repression), while that of RELA seemed to be increased (Fig. 6G). Confirming a previous report [65], well-established Müller cell homeostasis genes such as glutamine synthetase [10, 66–68] and Kir4.1 [69, 70] were expressed at higher levels upon cortisol treatment (Fig. 6H). No or moderate changes were observed for marker genes of rods (rhodopsin), cones (arrestin 3), synapses (HOMER1) or ganglion and amacrine cells (calretinin) (Fig. 6I). Accordingly, cortisol treatment may help to maintain Müller cells in their neuron-supportive state preventing gliosis induction, which is partially reflected by the stable or even slightly improved expression of neuronal markers (e.g. HOMER1). Immunostaining of explants at 2 days in vitro (DIV) confirm that glutamine synthetase expression seems enhanced in cortisol treated samples (Fig. 6J). To validate this finding, qPCR on purified Müller cells from retinal explant cultures at 2 DIV was performed and showed consistent upregulation of glutamine synthetase cortisol-treated samples (Fig. 6K).

**Fig. 7:**
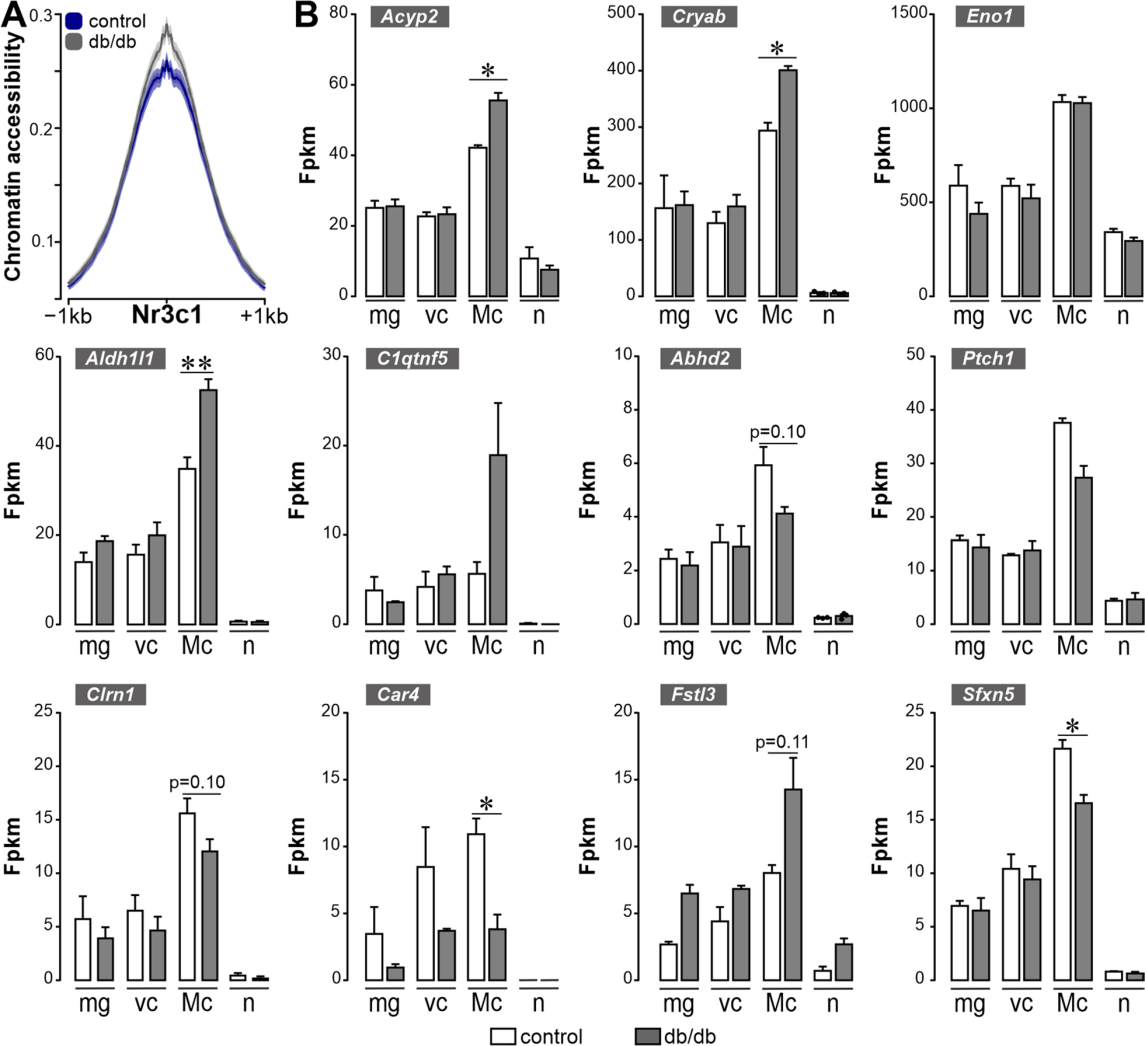
Regulation pattern of GR target genes in Müller cells of the diabetic mouse. **A** Accessibility at peaks associated with the GR (*Nr3c1*) motif is plotted comparing results from Müller cells isolated from 6-month-old control and db/db animals (*n* = 2 for each genotype). **B** Plots of transcript expression of GR target genes as determined by the RNA-seq experiment on purified retinal cell types from 6-month-old animals. Bars represent mean ± SEM from 3 biological replicates per genotype. Unpaired *t*-test: *p < 0.05; **p < 0.01

Having demonstrated Müller cell-specific expression and down-regulation of the GR in early DR and the finding that enhanced GR-mediated signalling promotes a potentially more neuron-supportive Müller cell phenotype in cortisol-treated retinal explants, we revisited our ATAC- and RNA-seq data to check whether the regulation pattern of known GR target genes is in line with our assumption that reduced GR activity is a key driver of Müller cell gliotic alterations. Confirming the data presented in Fig. 4, we found an enhanced accessibility at peaks associated with GR *(Nr3c1)* motifs in 6-months old diabetic animals (Fig. 7A). Next, we analysed whether the altered DNA accessibility at *Nr3c1* binding motifs is reflected by changes in mRNA levels of known GR target genes (Fig. 7B). The data of GR target gene expression and their regulation in Müller cells of diabetic retinae was rather heterogeneous. While a number of target genes (e.g. *Abhd2*, *Ptch1*, *Clrn1*, *Car1*, *Sfxn5*) are downregulated, suggesting that GR may act as a transcriptional activator, we also found that GR target genes, e.g. *Acyp2*, *Cryab*, *Aldh1l1*, *Fstl3*, that were up-regulated. This suggests that GR acts as an activator or repressor depending on the DNA landscape and potential interaction with additional transcription factors on these target genes in Müller cells.

### Therapeutic intervention by overexpression of GR specifically in Müller cells of db/db mice

**Fig. 8:**
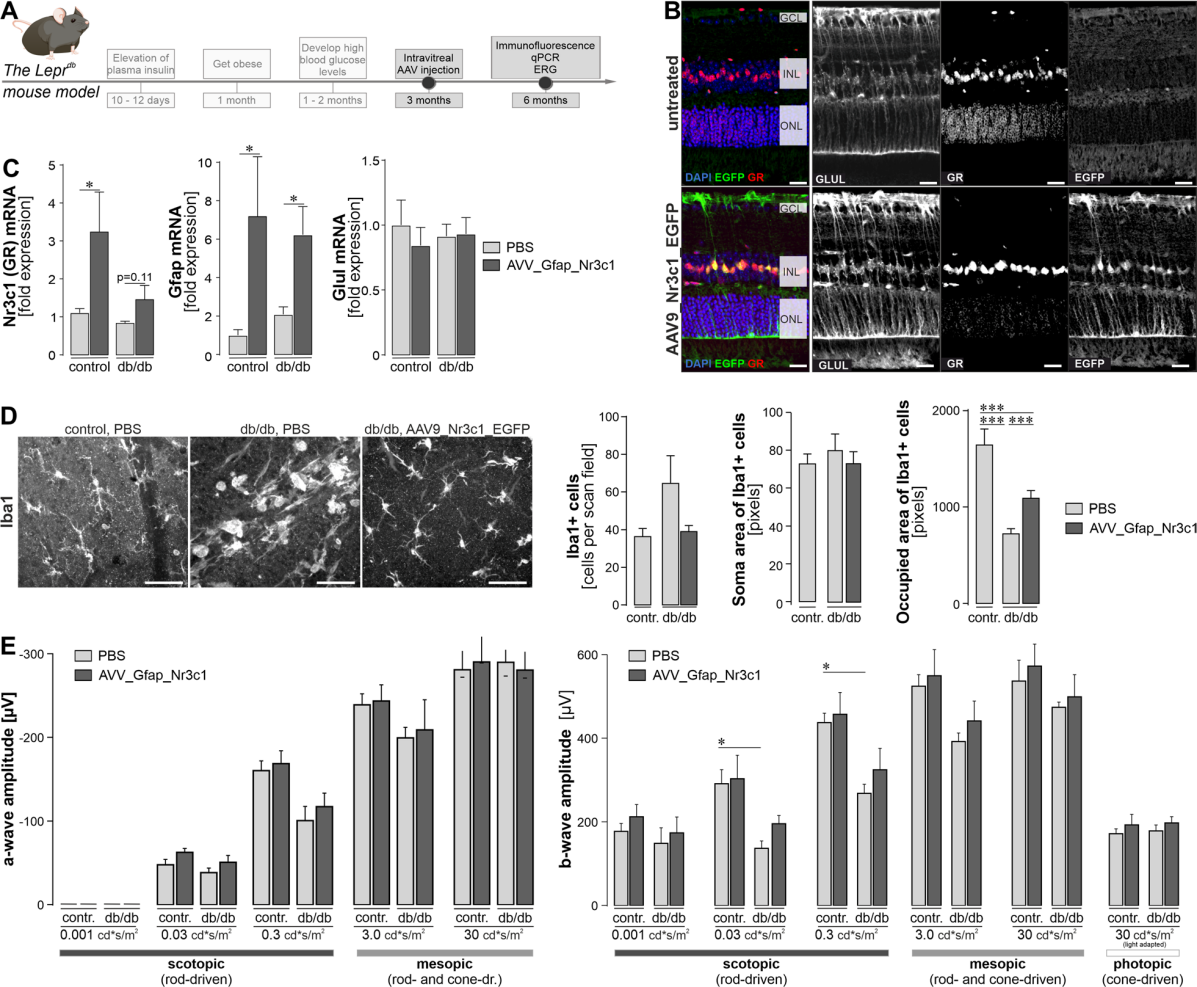
Proof of concept experiment investigating the impact of overexpressing GR (gene ID: Nr3c1) in Müller cells of the diabetic mouse retina in vivo. **A** Experimental design and the time line of treatment and readouts in relation to the development of diabetes in the db/db mice. **B** 2.5 × 10^13^ GC/ml AAV9-Nr3c1-EGFP particles were injected intravitreally in 3-month-old diabetic and control mice. Tissue was collected 3 months thereafter and stained for the Müller cell marker glutamine synthetase (GLUL). EGFP indicated successful viral transduction. *GCL*, ganglion cell layer; *INL*, inner nuclear layer; *ONL*, outer nuclear layer. Scale bars, 20 µm. **C** qPCR on MACS-sorted retinal cells isolated from 6-month-old mice, 3 months after single AAV injection, was performed. Only Müller cell-specific gene expression is shown. Bars represent mean values ± SEM. Biological replicates were as follows: *n* = 7 in controls and *n* = 5 for db/db mice. Unpaired *t*-test: *p < 0.05. **D** Assessment of microglial responses upon AAV-treatment reveals reduced microglial activation in retinae with Müller glia-specific GR overexpression. *Left*, Representative micrographs depicting the Iba1-staining in retinal flat mounts that were used to assess microglial morphological alterations. *Right*, Quantification of microglial cell numbers (bars represent the mean ± SEM from 2 mice per genotype and condition), soma size and the area occupied by their processes. For the latter two parameters, bars represent the mean ± SEM: *n* = 30 microglia in control retinae with PBS-sham injection; *n* = 102 microglia in db/db retinae with PBS-sham injection; *n* = 99 microglia in db/db retinae with AAV injection. These cells were analysed in retinal flatmounts from two mice per genotype and condition. The bigger the soma area and the smaller the occupied area, the more activated the microglia are. Ordinary one-way ANOVA with Dunnett's multiple comparisons test, ***p < 0.001. **E** Electroretinogram recordings were performed 3 months after AAV injection. Primarily rod (scotopic)- or cone-driven responses (photopic) or mixed responses were quantified. A-wave (reflects photoreceptor responses) and b-wave (representing inner retinal response e.g. by bipolar cells) were evaluated. Bars represent mean ± SEM of 5 animals per genotype. The right eye received the AAV injection, while the contralateral eye was injected with an equal volume of PBS as sham control. In both control and db/db animals, one PBS-sham-injected eye had to be excluded from analysis because of cataract formation or intraocular haemorrhage after surgery, resulting in the following number of biological replicates per genotype and treatment: *n* = 4 PBS-sham-injected eyes from control mice; *n* = 5 AAV-injected eyes from control mice; *n* = 4 PBS-sham-injected eyes from db/db animals; *n* = 5 AAV-injected eyes from db/db mice. Unpaired *t*-test: *p < 0.05

In the final set of experiments, we aimed to restore high levels of GR expression in Müller cells of the diabetic retina. To achieve this, we implemented AAV9-vectorized GR overexpression driven by an optimized human GFAP promotor in animals 3 months of age and thus approximately 1 after onset of T2D (Fig. 8A). The GFAP promoter was chosen to specifically activate GR expression in diseased tissue and to avoid the autoregulatory loop that down-regulates GR expression in the presence of high levels of corticosterone, as happens with endogenous GR. Immunostaining for the reporter EGFP in eyes one month after intravitreal injection of either AAV or PBS (sham control) confirmed the glial specificity of the construct (Fig. 8B). Importantly, a moderate up-regulation of GR was observed in GLUL-coexpressing Müller cell bodies in the INL (Fig. 8B). Note that no change in GR expression was observed in the nerve fibre/ganglion cell layer where astrocytes, potentially also targeted by the GFAP promoter-driven construct, reside (Fig. 8B). This suggests that the effects of the treatment are mainly due to GR overexpression in Müller cells and that the moderate GFAP expression in Müller cells is sufficient to allow transgene expression even in wild type retina.

Three months after AAV injection, a quantitative expression analysis via qPCR was performed on purified Müller cells. A significant upregulation of the GR (*Nr3c1*) was noted in AAV-injected controls and a trend towards upregulation in db/db mice (Fig. 8C). Note the slight *Gfap* upregulation as a consequence of AAV injection. This was still present irrespective of the genotype and was not observed in the PBS sham-injected eyes.  No significant effect was observed on the expression of glutamine synthetase (Glul) in response to any of treatment conditions (Fig. 8C).

We next asked whether this Müller cell-restricted upregulation of GR affects the microglial response. As in the initial validation of control versus db/db mice at 6 months of age, there was a trend toward slightly higher microglial numbers in the diabetic retina of eyes that received only PBS as a sham control compared to non-diabetic control eyes that also received PBS sham injections (Fig. 8D). With respect to microglial soma area size, no significant difference was observed. This confirms that microglia in the retina of 6-month-old mice are just about to get activated and did not yet develop the full-blown phenotype of activated microglia yet. Notably, microglia in sham-treated diabetic mice had significantly shorter processes and, thus, occupied much smaller territories compared to those treated with AAV_Gfap_Nr3c1 (Fig. 8D).

Finally, ERG measurements were performed to test whether GR overexpression not only alters the gene expression signature of Müller cells and microglial activation, and therewith the retinal tissue homeostasis, but also indirectly enhances neuronal function. Similar to our initial characterisation of untreated db/db mice (Fig. 2E), a significantly reduced light response (a- and b-wave) in the retinae of diabetic mice compared to controls was observed when analysing the PBS sham-injected eyes of each genotype (Fig. 8E). This drop in retinal light responsiveness in db/db mice was no longer significant after AAV-mediated GR overexpression in Müller cells compared to the AAV-injected eyes of control mice. The treatment effect of GR overexpression in Müller cells was most pronounced for rod-driven (scotopic) b-waves compared to cone-driven (photopic) responses in db/db mice (Fig. 8E).

## Discussion

### Early functional, but late onset of morphological changes of distinct cell types in the db/db retina

Currently, no animal model fully replicates all facets of the pathophysiology of DR properly. The re-evaluation of the retinal phenotype of our db/db strain ensured that we had captured the right time window for our multiomics approach designed to identify early glial changes before fundamental neurodegeneration has occurred. In the db/db animals, the onset of pericyte loss at 3 months of age has been described as a key feature of DR [40]. We observed a decrease in the number of PDGFRβ-positive pericytes in db/db retinae, but not in animals younger than 6 months of age. This alings with reports that BRB breakdown and increased apoptosis occur in older db/db mice [44, 45]. Similarily, while microglial activation was observed in mice aged 6-9 months, we detected pro-inflammatory microglial signatures in 6-month-old db/db mice before major cellular architecture changes, consistent with other studies [37, 49]. However, we detected typical pro-inflammatory microglial signatures in cells isolated from retinae of 6-months-old db/db mice similar to earlier reports [71], thus, before major changes in their cellular architecture occurred, which is supported by the results of other studies [72].

Little is known about changes of Müller cells in the course of DR progression in db/db mice. An upregulation of GFAP in Müller cells, as a marker of their gliotic activation, has been described in 2-month-old db/db mice [73]. In line with this, we demonstrate a moderate but significant upregulation of GFAP in 6-month-old animals via cell type-specific RNA-seq, qPCR, and proteomic data, whereas we were unable to detect the protein in Müller cell processes by immunolabeling. Our MACS approach has the limitation that Müller cells may be slightly contaminated with astrocytes, which express high levels of GFAP in the homeostatic retina. Thus, the increase in GFAP levels detected in the MACS-purified glial population may also be due to expression changes in astrocytes that has consistently been documented in db/db mice [37, 72, 74] or may be too low to be detected in Müller cells by immunostaining as also described by others [75]. However, other molecular markers of Müller cell gliosis were found to be dysregulated including Kir4.1, *Stat3*, *S100a1* and  Cd44 [47, 48]. Even more important, using physiological measurements, we were able to clearly demonstrate that Müller cell gliosis is present in the retinae of 6-month-old db/db animals at the functional level. As we have shown previously in diabetic rats [17, 76], also Müller cells from db/db mice had a reduced potassium conductance and consequently a diminished ability to compensate for osmotic stress. This finding implies that the Müller cells’ capacity to perform potassium syphoning, key for the retinal ion and volume homeostasis [12], is already perturbed in this early stage of DR. Thus, this could be one driving factor of disease progression as neuronal functional and retinal tissue integrity rely on this glial housekeeping function [13, 69].

Therefore, we also evaluated neuronal survival and function. Reports about morphological alterations of the db/db retina and the time course thereof are rather controversial. Bogdanov et al. [73] describe neurodegeneration as early as 2 months of age – an age when mice just develop hyperglycaemia. However, even though they show a slightly reduced retinal thickness at this early age, they do not show a loss of neuronal cells over the course of ageing from 2 to 6 months, which one would expect to see in a progressive disease like DR. More consistent with our findings, many other studies report no change in retinal layer thickness in db/db mice at approximately 3 months of age, but an onset of retinal thinning and neuronal cell loss at approximately 6–7 months of age [37, 44, 49]. We found significantly reduced cone numbers, which is in line with other studies that found predominant cone photoreceptor dysfunction in early-stage DR in zebrafish [77] and humans [78, 79]. Findings regarding functional changes in the retinae of db/db mice are much more consistent. In agreement with other reports [73, 80, 81], we were able to show a functional decline of db/db retinal neurons as determined by reduced a- and b-wave amplitudes in ERG recordings in 6-month-old mice. A possible underlying reason for these deficits in retinal signalling processing at the level of photoreceptors (a-wave) or bipolar cells (b-wave) might be the observed disturbed Müller glial homeostasis function which we aim to target with our glia-centric treatment approach before irreversible neuronal loss has occurred.

### Early changes in the expression landscape of Müller glia in the db/db retina

In order to identify targets for potential treatment, we determined the expression landscape of Müller cells the diabetic retina from 6-month old mice. Multiple changes as compared to cells from age-matched controls consistent with the observed functional alterations were observed in the glial mRNA and protein expression profiles. (i) The metabolism of the cells was significantly altered, with a reduction in triglyceride ligase expression, but enhanced expression of enzymes involved in mitochondrial beta-oxidation. (ii) Glial stress defence mechanisms were upregulated, such as glutathione transferase or aldehyde dehydrogenase activity that are key to aldehyde detoxification including protection from lipid peroxidation that is known to be elevated in diabetes because of enhanced levels of oxidative stress [82–84]. (iii) Signalling pathways, including those mediated by TGFβ, insulin-like growth factor-both via receptor tyrosine kinase-related mechanisms-and in addition G protein-mediated signalling in general, were upregulated in Müller cells from diabetic mice. The latter seems in agreement with a recent finding that modulation of G-protein-mediated signalling by application of phosphodiesterase inhibitors is beneficial in various models of retinal degeneration and enhances the tissue stress resilience [85]. (iv) Several potassium channels and transporters were downregulated especially at protein level. At the early stage of DR that we studied here, the overall response of Müller cells is well suited to preserve and protect the retinal tissue, although initial functional adaptations (e.g. reduced potassium conductance, impaired cell volume regulation, upregulation of GFAP) indicate that the cells potentially are on the verge of developing a less supportive, gliotic phenotype.

Previous bulk RNA-seq or proteomic approaches performed on 10–12 week-old db/db or Akimba (Ins2Akita × Vegfa ) mice, a type 1 diabetes model of DR, identified GO terms related to synaptic transmission, glutamate transport and mitochondrial genes as downregulated. Pathways related to a metabolic shift from glycolysis to oxidative phosphorylation, activation of microglia/macrophages, metal ion and oxidative stress response were upregulated transcripts [73, 86] or proteins [87], largely confirming our data. Kandpal et al. [88] investigated mRNA changes by whole retinal RNA-seq 8 months after diabetes induction in a streptozotocin model of type I diabetes in mice and found alteration in pathways such as inflammation, microvasculature remodelling, apoptosis, glucose metabolism, Wnt signalling and photoreceptor biology indicating a more advanced stage of disease. In addition, Grant et al. [89] showed that ischaemia-mediated overexpression of growth factors such as VEGF, insulin-like growth factor-1, angiopoietin-1 and -2, stromal-derived factor-1, fibroblast growth factor-2, and tumour necrosis factor occurs in DR. This is consistent with our finding that growth factor signalling pathways are upregulated in Müller cells.

However, when comparing our data with those of studies performed on whole retinal extracts, the observed discrepancies regarding differential regulation of genes involved in metabolism, homeostasis and inflammation could be due to a response of different cell types. Single cell RNA-seq (scRNA-seq) of retinal tissue from 3-month-old Akimba mice identified upregulation of genes associated with ribosome, cytoskeleton, immune system processes, S100 proteins, glutathione metabolism, iron ion homeostasis, cell cycle regulation/apoptosis, and oxidative phosphorylation (OXPHOS) networks in Müller cells/macroglia [86]. These findings align with our data from 6-month-old db/db mice. For example, genes involved in glutathione metabolism such as Gstm1 were upregulated in macroglia from Akimba mice as well as specifically in Müller cells in our diabetes model, indicating a consistent anti-stress response of the cells in both models. The same accounts for Aldh1l1, which was identified in the van Hove data set [86] and was also clearly up-regulated at transcript and protein level in our bulk data on Müller cells. Moreover, van Hove et al. [86] reported that genes involved in glycolysis, central nervous system development, and OXPHOS were downregulated in Akimba macroglia. Some of these genes were also significantly expressed by Müller cells, as well as down regulated in the diabetic retina in our data set: GUF1 homolog, GTPase (Guf1) is involved in metabolic processes of organo-nitrogen compounds (Additional file 1: Table S1). Fibroblast growth factor binding protein 3 (Fgfbp3) and phosphotyrosine interaction domain containing 1 (Pid1) contribute to the regulation of phosphate metabolism (Additional file 1: Table S1).

Importantly, our study differs from previously published work in that we provide cell type-specific insights into disease-associated expression profiles not only at the transcript but also, for the first time, at the protein level. The importance of this for delineating the functional change of a cell is underscored by the fact that we, like many others before us [90–92], found considerable discordance in the transcript and protein levels of distinct genes (e.g. YAP1)—so one cannot reliably interpolate from changes in transcript levels to how the corresponding protein expression will change. Since in most cases it is the protein that mediates the actual function of a (protein-coding) gene, our multi-layered omics approach offers a more comprehensive understanding of diabetes' impact on cellular interactions in the retina compared to studies focusing solely on mRNA analysis.

### The GR as potential master regulator of Müller cells in the diabetic retina

In our search for key regulators that drive Müller cell changes in DR progression that could then be targeted by a therapeutic approach, the GR emerged as highly promising candidate. It was the one of few TFs for which ATAC-seq performed on purified Müller cells identified a significant alteration in the accessibility of its DNA-binding motives. This aligned well with the oPOSSUM-3 TF binding site cluster analysis on the basis of the RNA-seq data of differentially expressed genes in Müller cells from db/db mice that, besides others, identified the GR as one of the most likely TFs that could be causative for the observed changes in gene expression profiles. Finally, only the GR fulfilled our additional screening criteria—a high expression in Müller cells and a differential expression in Müller cells of 6-month-old db/db mice at transcript and protein level. Importantly, we were able to clearly localise GR immunoreactivity to Müller cell nuclei, suggesting that it is active in the cells, which is consistent with the high corticosterone levels present especially in the blood plasma of db/db animals together with the increased level of GR phosphorylation determined by Western blot analysis. Furthermore, increased GR mRNA expression within 4 h after NMDA-induced damage, normalised to pre-damage levels by day 2 and below pre-damage levels by day 3, has also been described in the chicken retina [25]. A similar response, but with much longer time scales, may also be active during DR progression.

Even though recent studies suggest potential roles for the GR in DR, it is important to note that the understanding of exact mechanisms of the interplay between the GR, inflammation, vascular changes, neuroprotection, and other molecular pathways involved in the development and progression of DR are still being elucidated. DR is associated with chronic low-grade inflammation and immune dysregulation, which could also be confirmed in our present study. The GR has been implicated in regulating vascular permeability in the retina, e.g. by inhibiting VEGF-induced permeability in endothelial cells, potentially offering a protective effect against retinal vascular leakage [93]. In human retinal endothelial cells (HRECs), dexamethasone, a GR ligand, significantly reduced glucose-induced cell loss and vascular permeability, suggesting a potential role of GR in protecting this cell type in high glucose condition and facilitating endothelial cell repair in the diabetic retina [94, 95]. GR activation has been shown to promote neuronal survival and decrease apoptotic cell death, suggesting that it might protect retinal neurons from DR-related damage[96–98].

The major ligand of the GR in mice is the glucocorticoid corticosterone. Circulating levels of glucocorticoids are regulated by the hypothalamic–pituitary–adrenal (HPA) axis. During stress, the HPA axis is activated and an increase in glucocorticoids helps the body cope with and recover from the stressful situations [99]. As in our study, others have described elevated corticosterone levels in mouse models of type 1 and type 2 diabetes [100]. Similarly, patients with diabetes present with higher urinary free cortisol [28]. Chronic administration of glucocorticoids can constitutively downregulate GR expression via an autoregulatory loop [30]. Therefore, persistently high concentrations of corticosterone in the blood may negatively regulate GR expression in diabetic mice.

As a nuclear hormone receptor, GR coordinates inflammation, cell proliferation and differentiation in target tissues. Consistent with our findings, Gallina et al. [25] found that GR was mainly located in Sox2-positive nuclei of Müller cells in mouse, guinea pig, dog, and human retinae. Partial loss of retinal GR results in a thinner INL, further supporting its critical role in the maintenance of retinal homeostasis [101]. In addtion, Gallina et al. [31] found that activation of GR inhibited the reactivity of microglia and the loss of retinal neurons upon excitotoxic tissue damage. The activated GR can regulate the expression of target genes through multiple ways. It can lead to transactivation or transrepression of gene transcription directly by binding to glucocorticoid-response elements (GREs) in regulatory regions of specific target genes [95, 102]. An alternative mode of GR action is through protein–protein interactions with other TFs like the nuclear factor kappa-light-chain enhancer of activated B cells (NF-κB), activating protein-1 (AP-1) or YAP1 [95, 96, 102].

YAP1, for instance, is the central factor on which the Hippo pathway converges. In case the Hippo signalling pathway is less active, YAP1 translocates into the nucleus, followed by the activation of its downstream targets which are associated amongst others with increased proliferation and cell cycle entry [103, 104]. In line with this, inhibition of YAP1 signalling via the Hippo pathway prevents Müller cell proliferation upon injury in mammals whereas proliferation is induced via enhanced YAP1 activation [105, 106]. Interestingly, YAP1 seems to be co-regulated with GR [107, 108], which we could confirm at transcript level. However, at protein level, we detected higher YAP1. One reason could be that the high levels of glucose lead to production of uridin-5′-diphospho-N-acetylglucosamine (UDP-GlcNAc) and this results in increased YAP O-GlcNAcylation which can stabilise YAP protein levels [109, 110]. Therefore, the ultimate impact of changing GR levels on YAP1-mediated pathways should be investigated in more detail in future studies.

The GR target gene cluster identified by the oPOSSUM-3 TF analysis consisted mainly of downregulated genes. Interestingly, a quarter of the significantly downregulated genes in Müller cells of the db/db mouse retina were also potential target genes of the GR. One such example is the transcript of forkhead box o1 (*Foxo1*). It is involved in the control of insulin sensitivity, hepatic glucose production, and blood glucose levels [111]. Carbonic anhydrase 4 (*Ca4*), a member of a large family of zinc metalloenzymes that catalyses the reversible hydration of carbon dioxide, is another example of a significantly downregulated GR target gene in Müller cells of diabetic mice. *Ca4* is essential for acid removal from the retina, while mutations in *Ca4* impair pH regulation and cause retinal photoreceptor degeneration [112]. The strong impact of these two exemplary GR target genes on retinal integrity suggests that regulation of the GR target gene cluster in the diabetic retina has great potential to slow disease progression.

### AAV-vectored GR overexpression in Müller cells improves neuronal function in db/db mice

Treatment strategies for DR (e.g. anti-VEGF, intravitreal steroids, photocoagulation) require lifelong repeated invasive interventions with significant side effects. Given our findings that the GR is specifically regulated in Müller cells of the diabetic retina and that its activity has beneficial effects on the cells as well as on neighbouring neurons, we tested our hypothesis that the GR is a promising potential target for a novel gene therapeutic approach. We restricted GR overexpression to Müller cells in order to ensure its specific action in glial cells only and to leave retinal neurons unaltered, which may be less able to cope with exogenous gene overexpression. This also spared other ocular tissues, which typically experience unwarranted side effects such as the formation of lens cataracts with the continued use of intravitreal steroids [113]. After validating the successful overexpression of GR in Müller cells, we analysed the Müller glial response, but found no difference, e.g. in the upregulation of the gliosis marker GFAP, which was still slightly upregulated in AAV-injected eyes of both genotypes 3 months after injection. This persistent moderate gliotic response of the cells after transduction by the virus was not observed in sham-injected eyes receiving PBS only. This finding underscores the current debate in the field on the need for a thorough re-evaluation and optimization of the immunogenic potential of AAV constructs in order to increase the efficacy of gene therapy approaches [114, 115].

We found a moderate but significant effect on microglia upon overexpression of GR in Müller cells. As in the initial validation of the retinal phenotype in the 6-month-old db/db mice, we did not observe genotype-dependent differences in microglia number or soma area. However, we additionally analysed the area occupied by microglial processes, which shrinks upon microglial activation [116, 117]. Indeed, we observed a significant reduction of the microglial occupied area in the diabetic retina compared to controls, which was reverse by overexpression of GR in Müller cells. This is a first indication supporting the idea that GR activity in Müller cells may also influence their close cross-talk with microglia, which is central to modulate retinal immune homeostasis [60, 117–120].

Finally, we used ERG recordings as a highly sensitive readout to test for potential treatment effects on neuronal function, as we found significant alterations in db/db mice at 6 months of age, whereas we found very little morphological changes at this stage of the disease, so that treatment effects would have been difficult to assess by morphometry. We confirmed the significantly reduced retinal light responsiveness of PBS-sham-treated diabetic retinae when compared to PBS-sham-treated control eyes. Overexpression of GR in Müller cells resulted in a moderate improvement of the light response especially in the cells integrating the input from rod photoreceptors such as bipolar cells, as reflected by the most pronounced differences between b-waves measured under scotopic conditions.  Thus, inner retinal neurons, such as the aforementioned bipolar cells, but possibly also amacrine or ganglion cells, may benefit most from the changes associated with GR overexpression in Müller cells. This would align with our recent study showing that improved homeostatic functions of Müller cells, such as a better-maintained potassium conductance, are specifically relevant to this neuronal cell population [69, 117]. Our results are in agreement with a recent study showing that targeting only Müller cells can be an effective treatment for the diabetic retina. In this study, AAV was used to enhance expression of RLBP1, a key Müller cell protein that functions as a retinoid carrier critical for photoreceptor function in the visual cycle, leading to significant therapeutic results [121].

## Conclusion

We identified the GR as a promising candidate to be targeted by gene therapeutic approaches as we and others demonstrated (i) GR expression to be almost exclusive to Müller glia, in a highly conserved manner amongst warm-blooded vertebrates (i.e. retinae of chicks, mice, guinea pigs, dogs and humans), (ii) many TFs downstream or co-active with GR are specifically expressed in Müller glia in the retina, (iii) GR expression is significantly and specifically down-regulated in gliotic Müller cells of the diabetic mouse retina, (IV) the oPOSSUM TF binding site cluster analysis based on our RNA-seq data identified GR as one of few strong candidates to explain the diabetes-associated expression changes in Müller cells which was also confirmed by Atac-seq, (v) stimulating GR signalling using cortisol in retinal explant cultures fostered the expression of genes important for neuron-supportive Müller cell functions, (vi) transgenic mice receiving injections of the selective GR agonist TA 4 days prior to conditional Müller cell ablation presented with significantly higher Müller cell survival and if left untreated, loss of Müller cells caused photoreceptor degeneration, vascular leakage and intraretinal neovascularization – hallmarks of DR, (vii) previous studies have shown that TA reduces vascular leakage, inhibits the secretion of VEGF and prevents osmotic swelling of Müller cells and (viii) finally, treatment with GR agonists (e.g. TA) has proven to be effective in inflammatory diseases, DR included, as it counterbalances typical changes associated with DR such as breakdown of the blood retinal barrier or onset of neuroinflammation. Future studies are needed to optimise AAV-vectored GR delivery and to tune its activity levels in Müller glia to avoid potential adverse cellular responses to AAV-driven construct expression, with the overall goal of improving the therapeutic efficacy of the approach presented here with initial pilot data.

## Methods

### Animals

Db/wt heterozygous mice (BKS.Cg-Dock7m^+/+^Leprdb/J) were obtained from Jackson Laboratories (https://www.jax.org/strain/000642) and BKS-Lepr^db/db^/JOrlRj were obtained from Janvier Labs (https://www.janvier-labs.com/en/fiche_produit/diabetique_mouse/) and bred in our animal facility. All experiments were performed in accordance with European Community Council Directive 86/609/EEC and were approved by local authorities (ROB-55.2-2532.Vet_02_18_20). Animals had free access to water and food in a climate-controlled room with a 12-h light–dark cycle. Mice of both sexes at 3, 6, and 9 months of age were used for the experiments. The genotyping of the mice was performed based on the protocol previously published by [122] using the following primers: fw_i_-ATT AGA AGA TGT TTA CAT TTT GAT GGA AGG; fw_o_-TTG TTC CCT TGT TCT TAT ACC TAT TCT GA; rev_i_-GTC ATT CAA ACC ATA GTT TAG GTT TGT CTA; rev_o_-CTG TAA CAA AAT AGG TTC TGA CAG CAA C. PCR of DNA from wildtype mice revealed two bands at 610 and 264 bp, that from db/ + mice three bands at 610, 406, and 264 bp, and that from homozygous db/db mice two bands at 610 and 406 bp. Wild type and db/ + mice were used as controls as they do not develop the diabetes phenotype including the typical gain in weight. To ensure that the mild genetic heterogeneity potentially introduced by crossbreeding mice from two suppliers did not affect the severity of the gross phenotype, gain in weight indicating metabolic dysregulation and ERG responsiveness to delineate retinal dysfunction were compared. We could confirm that the mouse strains obtained from Jackson and Janvier laboratories presented with almost identical phenotypes and effect size because of homozygous loss of leptin receptor function (Additional file 1: Fig. S5).

### Corticosterone level measurement in mouse blood

A corticosterone ELISA kit (Abcam, Cambridge, UK) was used according to the manufacturer's recommendations to measure corticosterone levels in the blood plasma of five 6-month-old mice per genotype. Samples were collected in blood collection tubes, centrifuged at 3000*g* for 10 min at 4 °C, and stored at -20 °C. Blood plasma samples were used for ELISA at a dilution of 1:100.

### Retinal explant culture

Retinal explants from wild-type mice were cultured for two days. Mice were euthanatized, eyes enucleated, and retinae carefully removed. Explant cultures were placed in a 24-well plate containing 500 μl medium (DMEM/F-12, GlutaMAX™, 1:100 antibiotic–antimycotic; Thermofischer) per well. Retinae were placed in the centre of a Whatman^®^ Nuclepore™ track-etched membrane (Merck, Darmstadt, Germany) and covered with a drop of medium. Retinal explants were maintained at 37 °C in 5% CO_2_ with daily medium changes, while cortisol was added twice daily to maintain a constant high concentration of 500 ng/ml.

### Patch clamp recordings of single Müller cells

For whole-cell patch clamp experiments, cells were isolated as described above. The cells were stored at 4 °C in serum-free minimum essential medium until use within 4 h after cell isolation. Müller cells were identified according to their characteristic morphology. The currents were recorded at room temperature using the Axopatch 200A amplifier (Axon Instruments, Foster City, CA, USA) and the ISO-2 software (MFK, Niedernhausen, Germany). The signals were low-pass filtered at 1 or 6 kHz (eight-pole Bessel filter) and digitised at 5 or 30 kHz, respectively, using a 12-bit A/D converter. Patch pipettes were pulled from borosilicate glass (Science Products, Hofheim, Germany) and had resistances between 4 and 6 MΩ when filled with a solution containing (mM): 10 NaCl, 130 KCl, 1 CaCl_2_, 2 MgCl_2_, 10 EGTA, and 10 HEPES, adjusted to pH 7.1 with Tris. The recording chamber was continuously perfused with ECS. To evoke membrane currents, de- and hyperpolarizing voltage steps of 250 ms duration, with increments of 10 mV, were applied from a holding potential of − 80 mV. The amplitude of the steady-state inward currents was measured at the end of the 250-ms voltage step from − 80 to − 140 mV. The membrane capacitance of the cells was measured by the integral of the uncompensated capacitive artefact (filtered at 6 kHz) evoked by a 10-mV voltage step in the presence of extracellular BaCl_2_ (1 mM). Current densities were calculated by dividing inward current amplitudes evoked by 60 mV hyperpolarization by the membrane capacitance. The resting membrane potential was measured in the current-clamp mode. Data are expressed as mean ± standard deviation, significance was determined by the non-parametric Mann–Whitney U test.

### Measurement of Müller cell volume regulation

Volume changes in retinal Müller cells were measured as described [123]. Briefly, retinal slices were loaded with the vital dye Mitotracker Orange (10 µM, excitation: 543 nm, emission: 560 nm long-pass filter; Life Technologies), which is preferentially taken up by Müller cells [124]. Slices were exposed to hypotonic solution (60% of control osmolarity using distilled water) for 4 min. Somata of labelled Müller cells were imaged using confocal microscopy (custom-made VisiScope CSU-X1 confocal system equipped with high-resolution sCMOS camera; Visitron Systems, Puchheim, Germany) and their cross-sectional areas were measured (ImageJ).

### Histological and immunohistochemical staining

Enucleated eyes were immersion-fixed (4% paraformaldehyde (PFA) for 1 h, room temperature), washed with phosphate-buffered saline (PBS), cryoprotected in sucrose, embedded in Cryomatrix™ (Thermofisher, Germany) and cut in 20 µm sections using a cryostat. Similarly, retinal explant cultures were removed from the medium and were fixed still directly on culture inset with 4% PFA at for 1 h at room temperature. Retinal sections were permeabilized (0.3% Triton X-100 plus 1.0% DMSO in PBS) and blocked (3% DMSO, 0,1% Triton X-100, 5% goat oder donkey serum in PBS) for 1 h at room temperature. Primary antibodies (Table 1) were incubated overnight at 4 °C. Sections were washed (1% bovine serum albumin in PBS) and incubated with secondary antibodies (2 h at room temperature; Table 1). Cell nuclei were labelled with DAPI (1:1000; Life Technologies). Sections were mounted using Aqua-Poly/Mount (Polysciences Europe, Eppelheim, Germany). Retinal flat mounts were labelled using a similar protocol, except that tissue was permeabilized by higher concentrations of 2% BSA, 0.5% Triton-X 100 und 5% goat/donkey serum in PBS and secondary antibodies were also incubated at 4 °C overnight. Control experiments without primary antibodies showed no unspecific labelling. Images were taken with a custom-made VisiScope CSU-X1 confocal system (Visitron Systems, Puchheim, Germany) equipped with high-resolution sCMOS camera (PCO AG, Kehlheim, Germany). For cell number quantification, images of retinal sections or z-stacks of retinal flatmounts were taken scanning through the nerve fibre layer to the outer nuclear layer of flatmounts (optical section thickness: 1 µm). Scans were confined to the central retina in close proximity to the optic nerve head, and morphometric parameters were assessed using Fiji [125].

**Table 1:** Antibodies and antibody-conjugated beads used in the present study

Name	Supplier	Order number
*1° antibodies for immunofluorescence labelling and Western blot*		
Goat anti-calretinin	Swant	CG1
Goat anti-EGFP	Rockland	600-101-215
Mouse anti-glutamine synthetase	Millipore	MAB302
Mouse anti-GFAP	Sigma-Aldrich	G3893
Rabbit anti-cone arrestin	Millipore	AB15282
Rabbit anti-GR	Cell Signalling	12041
Rabbit anti-Iba1	Wako	019-19741
Rabbit anti-Pde6b	Thermo Fisher	PA1-722
Rabbit anti-PDGFRß	abcam	ab32570
Rabbit anti-phospho-GR	Thermo Fisher	PA5-17668
Rabbit anti-Pdhb	Abcam	ab155996
*2° antibodies for immunofluorescence labelling, STED and Western blot*		
Donkey IgG anti-Goat IgG-Alexa Fluor 647	Dianova	705-605-003
Donkey IgG anti-goat IgG-Cy2	Dianova	705-225-147
Donkey IgG anti-goat IgG-Cy3	Dianova	705-165-003
Donkey IgG anti-mouse IgG-Cy2	Dianova	715-225-150
Donkey Fab anti-mouse IgG-Cy3	Dianova	715-167-003
Donkey IgG anti-rabbit IgG-Cy5	Dianova	711-175-152
Goat IgG anti-mouse IgG-Cy3	Dianova	115-165-146
Donkey anti-rabbit IgG-Alexa Fluor 488	Invitrogen	A21206
Donkey anti-rabbit IgG-Alexa Fluor 555	Invitrogen	A31572
Goat IgG anti-rabbit IgG-Alexa Fluor 488	Dianova	111-545-144
Goat anti-rabbit IgG, chain specific peroxidase conjugate	Calbiochem	401315
Goat anti-mouse abberior STAR 580	Abberior	ST580-1001
Goat anti-rabbit abberior STAR 635P	Abberior	ST635P-1002
*Magnetic-activated cell sorting (MACS)*		
Anti-Biotin microbeads ultrapure	Miltenyi Biotec	130-105-637
CD11b microbeads, human, mouse	Miltenyi Biotec	130-093-634
CD29-biotin, mouse	Miltenyi Biotec	130-101-943
CD31 microbeads, mouse	Miltenyi Biotec	130-097-418

For super resolution microscopy, fixed retinal sections were permeabilized with Triton X-100 (0.5% in 2% BSA in PBS) for 2 h and incubated with primary antibodies in blocking solution overnight at 4 °C. After rinsing several times with PBS, sections were incubated with secondary antibodies (Abberior Star) and DAPI for 2 h in blocking solution at RT, subsequently rinsed 3 times with PBS and once with distilled water. Sections were mounted with ProLong Gold antifade reagent (Invitrogen, Life Technologies). Stimulated emission depletion (STED) microscopy was performed at the Core Facility Bioimaging of the Biomedical Center of the LMU München with an inverted Leica SP8 STED X WLL microscope using appropriate lasers for fluorophore excitation (405 nm; pulsed white light laser 470–670 nm). Image acquisition was performed with a 93x/1.3 NA glycerol immersion objective with the pixel size set to 32 nm. To avoid bleed-through, the colour channels were recorded sequentially. The following spectral settings were used: DAPI (excitation: 405 nm; emission: 415–471 nm; PMT), AbberiorStar 580 (580 nm; 590–620 nm; HyD; depletion laser: pulsed 775 nm, at 20% intensity), AbberiorStar 635P (635 nm; 645–702 nm; HyD; depletion laser: pulsed 775 nm, at 12% intensity). Image contrast and brightness was adjusted with the open source software FIJI (Schindelin et al., 2012).

### Trypsin digest

The vascular architecture of the retina was studied using a trypsin digest-based protocol as described recently [126]. Enucleated eyes were fixed in 4% PFA for 24 h at 4 °C before retinal isolation. The cornea and lens were removed, and the retina was carefully separated from the sclera and choroid. After five gentle shaking washes with sterile water at RT for 30 min, the retinae were left in water on the shaker at RT overnight. The water was removed and the retinae were digested with 3% trypsin in 0.1 M Tris buffer with gentle shaking at 37 °C for 1.5 h. The trypsin was replaced with sterile water and the internal limiting membrane was removed. The retinal vasculature was then separated from the rest of the tissue by a series of 5-min water washes. When little or no debris remained, the vascular network was carefully transferred to a microscope slide using a trypsin-coated, fire-polished glass pipette. After drying on a hot plate at 37 °C overnight, haematoxylin and eosin (H&E) staining was performed. The specimens were then mounted using Aqua-Poly/Mount (Polysciences Europe) and a custom-built VisiScope CSU-X1 confocal system (Visitron Systems) equipped with a bright field unit. The number of endothelial cells, pericytes, and acellular capillaries was assessed using the cellcounter tool of Fiji [125], and endothelial cells and pericytes were identified by their characteristic morphology (e.g. their nuclei) and their location on the vessel wall. The calculation of the endothelial cell/pericyte ratio is described by [40].

### Magnetic activated cell sorting

Different cell types of the retina were sequentially separated by magnetic activated cell sorting (MACS) as previously described [60]. Retinae were isolated and digested with 0.2 mg/ml papain in PBS/Glucose at 37 °C for 30 min. Afterwards, three washing steps with PBS/Glucose were performed and the tissue incubated with 200 U/ml DNase I at RT for 4 min. The retinae were dissociated in extracellular solution (ECS) to get a single retinal cell solution and centrifuged at 600 g and 4 °C for 10 min. The supernatant was removed and the cells were resuspended in ECS and incubated with CD11b (*Itgam*) microbeads (Table 1) which have been developed for the positive isolation of primary mouse CD11b^+^ microglia at 4 °C for 15 min. After centrifugation at 600*g* and 4 °C for 10 min the cells were resuspended in ECS and transferred onto a large cell column using a fire polished glass pipette. The cells were separated according to the manufacturer’s recommendation. The CD11b^+^ fraction was centrifuged and the cells stored at -80 °C. The CD11b^−^ cells (flow through) were centrifuged, resuspended in ECS and incubated with CD31 (*Pecam1*)-microbeads (Table 1) for the positive selection of CD31^+^ endothelial cells at 4 °C for 15 min. After centrifugation at 4 °C and 600 *g* for 10 min the cells were resuspended in ECS, transferred onto large cell columns and eluted according to the manufacturer’s recommendation. The CD31^+^ fraction was centrifuged and the cells stored at − 80 °C. The CD31^−^ cells (flow through) were centrifuged, resuspended in ECS and incubated with CD29 (*Itgb1*)-biotin at 4 °C for 15 min. The antibody binds to CD29 (Table 1), also known as integrin ß1, which is a cell-surface receptor expressed by Müller cells. Retinal astrocytes are assumed to be found in a small amount in the CD29^+^ fraction. The cells were centrifuged at 4 °C and 600*g* for 10 min, resolved in ECS and incubated with Anti-Biotin microbeads which enables the positive selection of the CD29^+^ Müller cells at 4 °C for 15 min. After centrifugation the cells were resuspended in ECS, transferred onto the columns and separated according to the manufacturer’s recommendation. The remaining CD29^−^ fraction mostly contained the retinal neurons. The remaining samples were centrifuged at 10,000*g* and 4 °C for 15 min and the cell pellet was stored at − 80 °C.

### qPCR

Total RNA was isolated from enriched cell populations using the PureLink^®^ RNA Micro Scale Kit (Thermo Fisher Scientific, Schwerte, Germany). A DNase digestion step was included to remove genomic DNA (Roche, Mannheim, Germany). First-strand cDNAs from 10 to 50 ng of total RNA were synthesised using the RevertAid H Minus First-Strand cDNA Synthesis Kit (Fermentas by Thermo Fisher Scientific, Schwerte, Germany). Primers were designed using the Universal ProbeLibrary Assay Design Center (Roche, Table 2). Transcript levels of candidate genes were measured by qPCR using cDNA with the QuantStudio 6 Flex Real-Time PCR System (Life Technologies, Darmstadt, Germany) according to the company’s guidelines.

**Table 2:** Primers used for qPCR

Primer	Sequence 5′3′
GFAP fw	TCG AGA TCG CCA CCT ACA G
GFAP rev	GTC TGT ACA GGA ATG GTG ATG C
Glul fw	GCC CAA GTG TGT GGA AGA G
Glul rev	AAG GGG TCT CGA AAC ATG G
Nr3c1 fw	TGA CGT GTG GAA GCT GTA AAG T
Nr3c1 rev	CAT TTC TTC CAG CAC AAA GGT
Pdhb fw	TTA AAT CGG CCA TTC GTG AT
Pdhb rev	CAG GAA ATC TTT TGA CTG AGC TT

### Western blot

Gels containing 12% acrylamide were used for sodium dodecyl sulphate–polyacrylamide gel electrophoresis (SDS-PAGE). Samples were mixed with 5× SDS-PAGE sample buffer, incubated at 95 °C for 10 min, and loaded onto the gel next to the prestained Protein™ ladder plus (Thermofisher Scientific, Darmstadt, Germany). Electrophoresis was performed at 60 V for 45 min and at 150 V for 70 min. The proteins were then transferred to a 0.22 µm PVDF membrane by blotting at 24 V for 35 min using a Trans-Blot Turbo™ system (BioRad, Feldkirchen, Germany). The membrane containing the proteins was blocked in 5% BSA in TBST at RT for 1 h to avoid unspecific binding of the antibodies. The expected size of GR was 95 kDa and that of phosphorylated GR was 86 kDa. PDHB was used as a housekeeping protein because it is present at similar levels in all retinal cell types. Since the expected size was 35 kDa and the molecular weight of the phospho-/GR was 86/95 kDa, the membranes were cut into two pieces at the 70 kDa band of the standard. The membrane pieces were shaken in the primary antibody overnight at 4 °C. The next day, they were washed three times in TBST for 10 min and incubated with the secondary antibody in 5% BSA in TBST at RT for 2 h. The Clarity Max™ Western ECL Substrate Kit (BioRad) was used and the bands were visualised using the ChemiDoc XRS + system (BioRad). GelAnalyzer 19.1 (www.gelanalyzer.com) was used to quantify the bands.

### Bulk-RNA sequencing

After immunoseparation of retinal cell types, total RNA was isolated from cell pellets using the PureLink^®^ RNA Micro Scale Kit (Thermo Fisher Scientific, Schwerte, Germany). Validation of RNA integrity and quantification was performed using the Agilent RNA 6000 Pico chip analysis according to the manufactures instructions (Agilent Technologies, Waldbronn, Germany). Enrichment of mRNA and library preparation (Nextera XT, Clontech), library quantification (KAPA Library Quantification Kit Illumina, Kapa Biosystems, Inc., Woburn, MA, USA) as well as sequencing on an Illumina platform (NextSeq 500 High Output Kit v2; 150 cycles) were performed at the service facility KFB Center of Excellence for Fluorescent Bioanalytics (Regensburg, Germany; www.kfb-regensburg.de). After de-multiplexing, a total of at least 20 million reads per sample was reached. Quality control of the reads and quantification of transcript abundance was performed with the Tuxedo suit, as described elsewhere [127, 128]. Briefly, adapter sequences were removed with cutadapt [129] and several quality control measures were queried with fastqc. No major problems with the sequencing data was detected. Next, the trimmed reads were aligned to the reference genome/transcriptome (mm10) with HISAT2 [130] and transcript abundance was estimated with stringtie, expressed as fragments per 1,000 base pairs of transcript per million reads (FPKM).

### ATAC-seq and data analysis

Müller cells were purified using the MACS protocol and subjected to the omni-ATAC protocol with slight modification [131]. Briefly, 100.000 viable, pelleted cells were resupended in cold ATAC resuspension buffer (10 mM Tris–HCl pH 7.5 (Invitrogen, catalog nr 15567027), 10 mM NaCl (Invitrogen, catalog nr AM9760G), 5 mM MgCl_2_ (Invitrogen, catalog nr AM9530G), 0.1% IgePAL (Sigma Aldrich, catalog nr I3021-50ML), 0.1% Tween-20 (Sigma Aldrich, catalog nr P9416-50ML), 0.01% Digitonin (Promega, catalog nr G9441)) and incubated on ice for 3 min. Lysed cells were washed with cold ATAC wash buffer (10 mM Tris–HCl pH 7.5, 10 mM NaCl, 5 mM MgCl_2_, 0.1% Tween-20) and nuclei pelleted 10 min at 500 g. Nuclei were resuspended in 25 µL of transposition mix (12.5 µl 2 × TD buffer, 1.25 µl transposase (Illumina, catalog nr 20,034,197), 8.25 µl PBS, 0.25 µl 1% Digitonin, 0.25ul 10% Tween-20, 2.5 µl nuclease-free water (Invitrogen, catalog nr AM9937)) and incubated at 37 ºC for 30 min, 1000 RPM. Transposed fragments were purified using a Zymo Clean and Concentrator5 kit (Zymo, catalog nr R1013), followed by amplification (Ultra II Q5, NEB, catalog nr M0544L) and double size selection of 100-1000 bp fragments with AMPure XP beads (Beckman Coulter, catalog nr A63881). Library was sequenced using 2 × 50 bp reads.

Differential accessible (DA) peaks were analysed using the csaw package [132], with loess normalisation. Within DA peaks, enriched motifs were identified using the Monalisa package [133].

### Label-free liquid chromatography mass spectrometry

MACS enriched retinal cell types from four control and diabetic mice at 24 weeks of age were collected as detailed in Pauly et al. [60] and proteolyzed with Lys-C and trypsin by a modified FASP procedure as described [134] resulting in two fractions after sequential proteolysis with first Lys-C (Wako Chemicals) followed by trypsin (Promega). Peptides were analysed on a Q Exactive HF mass spectrometer (Thermo Fisher Scientific) online coupled to a UItimate 3000 RSLC nano-HPLC (Dionex). Samples were automatically injected and loaded onto the C18 trap cartridge and after 5 min eluted and separated on the C18 analytical column (Acquity UPLC M-Class HSS T3 Column, 1.8 μm, 75 μm × 250 mm; Waters) by a 90 min non-linear acetonitrile gradient at a flow rate of 250 nl/min. MS spectra were recorded at a resolution of 60,000 with an AGC target of 3 × 1e6 and a maximum injection time of 30 ms from 300 to 1500 *m*/*z*. From the MS scan, the 10 most abundant peptide ions were selected for fragmentation via HCD with a normalised collision energy of 27, an isolation window of 1.6 *m*/*z*, and a dynamic exclusion of 30 s. MS/MS spectra were recorded at a resolution of 15,000 with a AGC target of 1e5 and a maximum injection time of 50 ms. Unassigned charges, and charges of + 1 and > 8 were excluded from precursor selection.

Acquired raw data were loaded to the Progenesis QI software for MS1 intensity based label-free quantification (Nonlinear Dynamics, Waters), separately for the two different proteolyzed fractions and for each cell type. After alignment in order to reach a maximum overlay of peptide features, filtering of singly charged features and features with charges > 7 and normalisation to correct for systematic experimental variation, all MSMS spectra were exported and searched against the Swissprot mouse database (16772 sequences, Release 2016_02) using the Mascot search engine. Search settings were: enzyme trypsin or Lys-C, respectively, 10 ppm peptide mass tolerance and 0.02 Da fragment mass tolerance, one missed cleavage allowed, carbamidomethylation was set as fixed modification, methionine oxidation and asparagine or glutamine deamidation were set as variable modifications. A Mascot-integrated decoy database search calculated an average false discovery of < 1% when searches were performed with a mascot percolator score cutoff of 13 and significance threshold of 0.05. Peptide assignments were re-imported into the Progenesis QI software. The two datasets per cell type were combined and the abundances of the top three intense unique peptides allocated to each protein were summed up. Missing values were imputed by low abundance imputation in Perseus. The resulting normalised abundances of the single proteins were then used for calculation of fold changes of proteins and significance values by a Student's *t*-test (based on log transformed values).

A principal component analysis of the proteomics data was performed and a heatmap which was focussing on proteins differentially regulated in Müller cells of diabetic mice generated by filtering *p* < 0.05 and at least twofold difference was performed via the R Core Team 2020. The effect of cortisol treatment on retinal explants was investigated in six retinae per treatment group. Samples were processed as described before. A principal component analysis of the proteomes was performed and a heatmap generated by filtering *p* < 0.05 and at least one, threefold difference. Pathway enrichment analysis for molecular functions for both data sets was done via the open-access platform PANTHER (released 20221013).

### Comparison of the two OMICS data sets

Since we acquired proteomic as well as transcriptomic data from 24-week-old db/db and wild-type mice in an analogous manner, we were interested in investigating how well changes in transcript translated to changes in protein. Briefly, FPKM or normalised abundance values were log-transformed, before a t-test between the respective glial and flow through values was calculated. Only genes/proteins with *p* < 0.05 and a glia:flow through ratio of twofold were considered to be glia-specific. The final comparison encompassed genes/proteins that were glia-specific in at least one of the two datasets and detected in both. Further, we calculated the ratios between the respective wild-type and mutant 6-month-old animals for each dataset enabling us to directly compare the inter-genotype differences between datasets. Finally, we calculated a spearman correlation coefficient. This analysis and the corresponding scatter plots were done using the R programming language (R Core Team 2014).

### Electroretinogram recordings (ERGs)

Mice were dark adapted overnight and the ERG measurements performed in the morning under dim red light illumination. The mice were anaesthetised with 100 mg/kg ketamine and 5 mg/kg xylazine by i.p. injection. The pupils of the mice were fully dilated with 0.5% tropicamide-phenylephrine 2.5% eye drops. Body temperature was maintained at 37 °C using a heating pad. Espion ERG Diagnosys equipment was used for the simultaneously recordings of both eyes. Corneal hydration was ensured by application of Methocel^®^ 2% eyedrops. A reference and a ground needle electrode were positioned subcutaneously between the eyes and in the back, respectively. Golden loop electrodes were placed on the cornea. Rod-driven responses were measured and quantified at scotopic light conditions (0.001 cd*s/m^2^). Furthermore, mixed (rod- and cone-driven) responses were analysed by applying light flashes of 3 cd*s/m^2^. Whereas cone-driven light responses (30 cd*s/m^2^) were recorded after 5 min of light adaption. Analysis of the data was done by using the Espion V6 software. A-wave amplitude was measured from baseline to the trough of the a-wave. B-wave amplitude was calculated from the trough of the a-wave to the peak of the b-wave.

### Intravitreal AAV administration

12-week-old mice were anaesthetised with 100 mg/kg ketamine and 5 mg/kg xylazine by intraperitoneal injection. After the pupils of the mice were fully dilated with 0,5% tropicamide-phenylephrine 2,5% eye drops, 2 µl of AAV9-GFAP(0.7)-mNR3C1-2A-EGFP particles (5 × 10^13^ GC/ml; Vector Biolabs, AAV-266053) or PBS (sham control) were administered using the NanoFil™ injection system from World Precision Instruments (WPI). The AAV9-GFAP(0.7)-mNR3C1-2A-EGFP vector enables mNR3C1 and EGFP expression driven by the GFAP promoter with a 2A linker in between for co-expression. Methocel^®^ 2% eyedrops were applied to the eyes of the mice after injection. 12 weeks post injection, morphometric analysis and ERG measurements were performed.

### Statistical analysis

The data were analysed with GraphPad Prism 8.4.0 (GraphPad Software, San Diego, CA, USA) and reported as mean ± standard error (SEM). Identification of outliers was performed with GraphPad PRISM 8. If not stated otherwise, statistical differences between two groups were compared with unpaired two-tailed Student *t* test. *p* < 0.05 was considered statistically significant.

### Supplementary Information

**Additional file 1**. Supplementary information provides more extensive description details of various studies on the used db/db mouse strain, focusing on the validation of vascular alterations (**Fig. S1**), validadtion of MACS-based enrichment effectiveness and glial activation analysis using RNAseq and mass spectrometry (**Fig. S2**), transcriptome and proteome comparison in Müller cells from diabetic retinas, highlighting concordant expression patterns (**Fig. S3**), unprocessed Western blots for GR and PDHB analysis in different cell types and conditions (**Fig. S4**) and a phenotypic comparison regarding body weight and ERG recordings of db/db mice from different laboratory backgrounds. 

## Data Availability

All data supporting the results of this study are available in the article and its supplementary information. ATAC-seq and RNA-seq data have been deposited in the Gene Expression Omnibus (GEO) database under accession number GSE236627. The mass spectrometry proteomics data have been deposited to the ProteomeXchange Consortium via the PRIDE [135] partner repository with the dataset identifier PXD045085.

## Abbreviations

AAV: Adeno-associated virus
ATAC-seq: Assay for transposase-accessible chromatin using sequencing
DR: Diabetic retinopathy
EGFP: Enhanced green fluorescent protein
ELISA: Enzyme-linked immunosorbent assay
ERG: Electroretinogram
GCL: Ganglion cell layer
GFAP: Glial fibrillary acidic protein
GR: Glucocorticoid receptor
INL: Inner nuclear layer
IPL: Inner plexiform layer
MACS: Magnetic activated cell sorting
ONL: Outer nuclear layer
OPL: Outer plexiform layer
OXPHOS: Oxidative phosphorylation
PBS: Phosphate-buffered saline
qPCR: Quantitative real-time polymerase chain reaction
TGFβ: Tumour growth factor β
VEGF: Vascular endothelial growth factor

## References

[1] Cheung N, Mitchell P, Wong TY (2010). Diabetic retinopathy. Lancet.

[2] Wang W, Lo ACY (2018). Diabetic retinopathy: pathophysiology and treatments. Int J Mol Sci.

[3] Sohn EH, van Dijk HW, Jiao C, Kok PH, Jeong W, Demirkaya N (2016). Retinal neurodegeneration may precede microvascular changes characteristic of diabetic retinopathy in diabetes mellitus. Proc Natl Acad Sci USA.

[4] Brooks HL, Caballero S, Newell CK, Steinmetz RL, Watson D, Segal MS (2004). Vitreous levels of vascular endothelial growth factor and stromal-derived factor 1 in patients with diabetic retinopathy and cystoid macular edema before and after intraocular injection of triamcinolone. Arch Ophthalmol.

[5] Itakura H, Akiyama H, Hagimura N, Doi H, Tanaka T, Kishi S (2006). Triamcinolone acetonide suppresses interleukin-1 beta-mediated increase in vascular endothelial growth factor expression in cultured rat Muller cells. Graefes Arch Clin Exp Ophthalmol..

[6] Wurm A, Iandiev I, Hollborn M, Wiedemann P, Reichenbach A, Zimmermann H (2008). Purinergic receptor activation inhibits osmotic glial cell swelling in the diabetic rat retina. Exp Eye Res.

[7] Wirostko B, Wong TY, Simo R (2008). Vascular endothelial growth factor and diabetic complications. Prog Retin Eye Res.

[8] Brownlee M (2001). Biochemistry and molecular cell biology of diabetic complications. Nature.

[9] Pitale PM, Gorbatyuk MS (2022). Diabetic retinopathy: from animal models to cellular signaling. Int J Mol Sci.

[10] Bringmann A, Grosche A, Pannicke T, Reichenbach A (2013). GABA and glutamate uptake and metabolism in retinal glial (Muller) cells. Front Endocrinol (Lausanne).

[11] Wurm A, Pannicke T, Iandiev I, Francke M, Hollborn M, Wiedemann P (2011). Purinergic signaling involved in Muller cell function in the mammalian retina. Prog Retin Eye Res.

[12] Bringmann A, Pannicke T, Grosche J, Francke M, Wiedemann P, Skatchkov SN (2006). Muller cells in the healthy and diseased retina. Prog Retin Eye Res.

[13] Reichenbach A, Bringmann A (2013). New functions of Muller cells. Glia.

[14] Shen W, Fruttiger M, Zhu L, Chung SH, Barnett NL, Kirk JK, Lee S, Coorey NJ, Killingsworth M, Sherman LS, Gillies MC. Conditional Müllercell ablation causes independent neuronal and vascular pathologies in a novel transgenic model. J Neurosci. 2012;32(45):15715–27. 10.1523/JNEUROSCI.2841-12.2012 10.1523/JNEUROSCI.2841-12.2012 PMC4014009 23136411

[15] Li Q, Puro DG (2002). Diabetes-induced dysfunction of the glutamate transporter in retinal Muller cells. Invest Ophthalmol Vis Sci.

[16] Lieth E, LaNoue KF, Antonetti DA, Ratz M (2000). Diabetes reduces glutamate oxidation and glutamine synthesis in the retina. The Penn State Retina Research Group. Exp Eye Res.

[17] Pannicke T, Iandiev I, Wurm A, Uckermann O, vom Hagen F, Reichenbach A (2006). Diabetes alters osmotic swelling characteristics and membrane conductance of glial cells in rat retina. Diabetes.

[18] Bringmann A, Pannicke T, Uhlmann S, Kohen L, Wiedemann P, Reichenbach A (2002). Membrane conductance of Muller glial cells in proliferative diabetic retinopathy. Can J Ophthalmol.

[19] Baumann B, Sterling J, Song Y, Song D, Fruttiger M, Gillies M (2017). Conditional Muller cell ablation leads to retinal iron accumulation. Invest Ophthalmol Vis Sci.

[20] Maharaj AS, Walshe TE, Saint-Geniez M, Venkatesha S, Maldonado AE, Himes NC (2008). VEGF and TGF-beta are required for the maintenance of the choroid plexus and ependyma. J Exp Med.

[21] Ford KM, Saint-Geniez M, Walshe T, Zahr A, D'Amore PA (2011). Expression and role of VEGF in the adult retinal pigment epithelium. Invest Ophthalmol Vis Sci.

[22] Dossarps D, Bron AM, Koehrer P, Aho-Glele LS, Creuzot-Garcher C, Net F (2015). Endophthalmitis after intravitreal injections: incidence, presentation, management, and visual outcome. Am J Ophthalmol..

[23] Das A, Stroud S, Mehta A, Rangasamy S (2015). New treatments for diabetic retinopathy. Diabetes Obes Metab.

[24] Semeraro F, Morescalchi F, Cancarini A, Russo A, Rezzola S, Costagliola C (2019). Diabetic retinopathy, a vascular and inflammatory disease: therapeutic implications. Diabetes Metab.

[25] Gallina D, Zelinka C, Fischer AJ (2014). Glucocorticoid receptors in the retina, Muller glia and the formation of Muller glia-derived progenitors. Development.

[26] Schaaf MJ, Cidlowski JA (2002). Molecular mechanisms of glucocorticoid action and resistance. J Steroid Biochem Mol Biol.

[27] Yeager MP, Pioli PA, Guyre PM (2011). Cortisol exerts bi-phasic regulation of inflammation in humans. Dose Response.

[28] Roy MS, Roy A, Brown S (1998). Increased urinary-free cortisol outputs in diabetic patients. J Diabetes Complicat.

[29] Chiodini I, Adda G, Scillitani A, Coletti F, Morelli V, Di Lembo S (2007). Cortisol secretion in patients with type 2 diabetes: relationship with chronic complications. Diabetes Care.

[30] Vandevyver S, Dejager L, Libert C (2014). Comprehensive overview of the structure and regulation of the glucocorticoid receptor. Endocr Rev.

[31] Gallina D, Zelinka CP, Cebulla CM, Fischer AJ (2015). Activation of glucocorticoid receptors in Muller glia is protective to retinal neurons and suppresses microglial reactivity. Exp Neurol.

[32] Shen W, Lee SR, Araujo J, Chung SH, Zhu L, Gillies MC (2014). Effect of glucocorticoids on neuronal and vascular pathology in a transgenic model of selective Muller cell ablation. Glia.

[33] Chen H, Charlat O, Tartaglia LA, Woolf EA, Weng X, Ellis SJ, Lakey ND, Culpepper J, Moore KJ, Breitbart RE, Duyk GM, Tepper RI, Morgenstern JP. Evidence that the diabetes gene encodes the leptin receptor: identification of a mutation in the leptin receptor gene in db/db mice. Cell. 1996;84(3):491–5. 10.1016/s0092-8674(00)81294-5. 10.1016/s0092-8674(00)81294-5 8608603

[34] Hammer SS, Vieira CP, McFarland D, Sandler M, Levitsky Y, Dorweiler TF, Lydic TA, Asare-Bediako B, Adu-Agyeiwaah Y, Sielski MS, Dupont M, Longhini AL, Li Calzi S, Chakraborty D, Seigel GM, Proshlyakov DA, Grant MB, Busik JV. Fasting and fasting-mimicking treatment activate SIRT1/LXRα and alleviate diabetes-induced systemic and microvascular dysfunction. Diabetologia. 2021;64(7):1674–89. 10.1007/s00125-021-05431-5. 10.1007/s00125-021-05431-5 PMC8236268 33770194

[35] Coleman DL, Hummel KP (1974). Hyperinsulinemia in pre-weaning diabetes (db) mice. Diabetologia.

[36] Hummel KP, Dickie MM, Coleman DL (1966). Diabetes, a new mutation in the mouse. Science.

[37] Majimbi M, McLenachan S, Nesbit M, Chen FK, Lam V, Mamo J (2023). In vivo retinal imaging is associated with cognitive decline, blood–brain barrier disruption and neuroinflammation in type 2 diabetic mice. Front Endocrinol (Lausanne).

[38] Kjorholt C, Akerfeldt MC, Biden TJ, Laybutt DR (2005). Chronic hyperglycemia, independent of plasma lipid levels, is sufficient for the loss of beta-cell differentiation and secretory function in the db/db mouse model of diabetes. Diabetes.

[39] Tonra JR, Ono M, Liu X, Garcia K, Jackson C, Yancopoulos GD (1999). Brain-derived neurotrophic factor improves blood glucose control and alleviates fasting hyperglycemia in C57BLKS-Lepr(db)/lepr(db) mice. Diabetes.

[40] Midena E, Segato T, Radin S, di Giorgio G, Meneghini F, Piermarocchi S (1989). Studies on the retina of the diabetic db/db mouse. I. Endothelial cell-pericyte ratio. Ophthalmic Res.

[41] Hanaguri J, Yokota H, Watanabe M, Yamagami S, Kushiyama A, Kuo L (2021). Retinal blood flow dysregulation precedes neural retinal dysfunction in type 2 diabetic mice. Sci Rep.

[42] Xiao C, He M, Nan Y, Zhang D, Chen B, Guan Y (2012). Physiological effects of superoxide dismutase on altered visual function of retinal ganglion cells in db/db mice. PLoS ONE.

[43] Li J, Wang JJ, Yu Q, Chen K, Mahadev K, Zhang SX (2010). Inhibition of reactive oxygen species by Lovastatin downregulates vascular endothelial growth factor expression and ameliorates blood-retinal barrier breakdown in db/db mice: role of NADPH oxidase 4. Diabetes.

[44] Yang Q, Xu Y, Xie P, Cheng H, Song Q, Su T (2015). Retinal neurodegeneration in db/db mice at the early period of diabetes. J Ophthalmol.

[45] Cheung AK, Fung MK, Lo AC, Lam TT, So KF, Chung SS (2005). Aldose reductase deficiency prevents diabetes-induced blood-retinal barrier breakdown, apoptosis, and glial reactivation in the retina of db/db mice. Diabetes.

[46] Shin ES, Sorenson CM, Sheibani N (2014). Diabetes and retinal vascular dysfunction. J Ophthalmic Vis Res.

[47] Roesch K, Stadler MB, Cepko CL (2012). Gene expression changes within Muller glial cells in retinitis pigmentosa. Mol Vis.

[48] Sigurdsson D, Grimm C (2023). Single-cell transcriptomic profiling of muller glia in the rd10 retina. Adv Exp Med Biol.

[49] Chen K, Wang Y, Huang Y, Liu X, Tian X, Yang Y (2023). Cross-species scRNA-seq reveals the cellular landscape of retina and early alterations in type 2 diabetes mice. Genomics.

[50] Lee ES, Lee JY, Jeon CJ (2010). Types and density of calretinin-containing retinal ganglion cells in mouse. Neurosci Res.

[51] Lee SC, Weltzien F, Madigan MC, Martin PR, Grunert U (2016). Identification of AII amacrine, displaced amacrine, and bistratified ganglion cell types in human retina with antibodies against calretinin. J Comp Neurol.

[52] Kern TS, Berkowitz BA (2015). Photoreceptors in diabetic retinopathy. J Diabetes Investig.

[53] Lyu Q, Xu S, Lyu Y, Choi M, Christie CK, Slivano OJ (2019). SENCR stabilizes vascular endothelial cell adherens junctions through interaction with CKAP4. Proc Natl Acad Sci USA.

[54] Ghinia Tegla MG, Buenaventura DF, Kim DY, Thakurdin C, Gonzalez KC, Emerson MM. OTX2 represses sister cell fate choices in the developing retina to promote photoreceptor specification. Elife. 2020;9. 10.7554/eLife.54279 PMC7237216 32347797

[55] Grosche A, Hauser A, Lepper MF, Mayo R, von Toerne C, Merl-Pham J (2016). The proteome of native adult muller glial cells from murine retina. Mol Cell Proteomics.

[56] Ho Sui SJ, Fulton DL, Arenillas DJ, Kwon AT, Wasserman WW. oPOSSUM: integrated tools for analysis of regulatory motif over-representation. Nucleic Acids Res. 2007;35(Web Server issue):W245-52. 10.1093/nar/gkm427. 10.1093/nar/gkm427 PMC1933229 17576675

[57] Kwon AT, Arenillas DJ, Worsley Hunt R, Wasserman WW (2012). oPOSSUM-3: advanced analysis of regulatory motif over-representation across genes or ChIP-Seq datasets. G3 (Bethesda)..

[58] Langouet M, Jolicoeur C, Javed A, Mattar P, Gearhart MD, Daiger SP (2022). Mutations in BCOR, a co-repressor of CRX/OTX2, are associated with early-onset retinal degeneration. Sci Adv.

[59] Kaufman ML, Goodson NB, Park KU, Schwanke M, Office E, Schneider SR, et al. Initiation of Otx2 expression in the developing mouse retina requires a unique enhancer and either Ascl1 or Neurog2 activity. Development. 2021;148(12). 10.1242/dev.199399 PMC8254865 34143204

[60] Pauly D, Agarwal D, Dana N, Schafer N, Biber J, Wunderlich KA (2019). Cell-type-specific complement expression in the healthy and diseased retina. Cell Rep.

[61] Davis CA, Hitz BC, Sloan CA, Chan ET, Davidson JM, Gabdank I, Hilton JA, Jain K, Baymuradov UK, Narayanan AK, Onate KC, Graham K, Miyasato SR, Dreszer TR, Strattan JS, Jolanki O, Tanaka FY, Cherry JM. The Encyclopedia of DNA elements (ENCODE): data portal update. Nucleic Acids Res. 2018;46(D1):D794-D801. 10.1093/nar/gkx1081. 10.1093/nar/gkx1081 PMC5753278 29126249

[62] Mathelier A, Zhao X, Zhang AW, Parcy F, Worsley-Hunt R, Arenillas DJ, Buchman S, Chen CY, Chou A, Ienasescu H, Lim J, Shyr C, Tan G, Zhou M, Lenhard B, Sandelin A, Wasserman WW. JASPAR 2014: an extensively expanded and updated openaccess database of transcription factor binding profiles. Nucleic Acids Res. 2014;42(Database issue):D142-7. 10.1093/nar/gkt997. 10.1093/nar/gkt997 PMC3965086 24194598

[63] Rouillard AD, Gundersen GW, Fernandez NF, Wang Z, Monteiro CD, McDermott MG, Ma'ayan A. The harmonizome: a collection of processed datasets gathered to serve and mine knowledge about genes and proteins. Database (Oxford). 2016;2016:baw100. 10.1093/database/baw100. 10.1093/database/baw100 PMC4930834 27374120

[64] Macosko EZ, Basu A, Satija R, Nemesh J, Shekhar K, Goldman M (2015). Highly parallel genome-wide expression profiling of individual cells using nanoliter droplets. Cell.

[65] Toops KA, Berlinicke C, Zack DJ, Nickells RW (2012). Hydrocortisone stimulates neurite outgrowth from mouse retinal explants by modulating macroglial activity. Invest Ophthalmol Vis Sci.

[66] Bringmann A, Pannicke T, Biedermann B, Francke M, Iandiev I, Grosche J (2009). Role of retinal glial cells in neurotransmitter uptake and metabolism. Neurochem Int.

[67] Poitry S, Poitry-Yamate C, Ueberfeld J, MacLeish PR, Tsacopoulos M (2000). Mechanisms of glutamate metabolic signaling in retinal glial (Muller) cells. J Neurosci.

[68] Reynisson H, Kalloniatis M, Fletcher EL, Shivdasani MN, Nivison-Smith L (2023). Loss of Muller cell glutamine synthetase immunoreactivity is associated with neuronal changes in late-stage retinal degeneration. Front Neuroanat.

[69] Pannicke T, Frommherz I, Biedermann B, Wagner L, Sauer K, Ulbricht E (2014). Differential effects of P2Y1 deletion on glial activation and survival of photoreceptors and amacrine cells in the ischemic mouse retina. Cell Death Dis.

[70] Reichenbach A, Wurm A, Pannicke T, Iandiev I, Wiedemann P, Bringmann A (2007). Muller cells as players in retinal degeneration and edema. Graefes Arch Clin Exp Ophthalmol.

[71] Kinuthia UM, Wolf A, Langmann T (2020). Microglia and inflammatory responses in diabetic retinopathy. Front Immunol.

[72] Arroba AI, Alcalde-Estevez E, Garcia-Ramirez M, Cazzoni D, de la Villa P, Sanchez-Fernandez EM (2016). Modulation of microglia polarization dynamics during diabetic retinopathy in db/db mice. Biochim Biophys Acta.

[73] Bogdanov P, Corraliza L, Villena JA, Carvalho AR, Garcia-Arumi J, Ramos D (2014). The db/db mouse: a useful model for the study of diabetic retinal neurodegeneration. PLoS ONE.

[74] Church KA, Rodriguez D, Mendiola AS, Vanegas D, Gutierrez IL, Tamayo I (2023). Pharmacological depletion of microglia alleviates neuronal and vascular damage in the diabetic CX3CR1-WT retina but not in CX3CR1-KO or hCX3CR1(I249/M280)-expressing retina. Front Immunol.

[75] Azrad-Leibovich T, Zahavi A, Gohas MF, Brookman M, Barinfeld O, Muhsinoglu O (2022). Characterization of diabetic retinopathy in two mouse models and response to a single injection of anti-vascular endothelial growth factor. Int J Mol Sci.

[76] Krugel K, Wurm A, Pannicke T, Hollborn M, Karl A, Wiedemann P (2011). Involvement of oxidative stress and mitochondrial dysfunction in the osmotic swelling of retinal glial cells from diabetic rats. Exp Eye Res.

[77] Alvarez Y, Chen K, Reynolds AL, Waghorne N, O'Connor JJ, Kennedy BN (2010). Predominant cone photoreceptor dysfunction in a hyperglycaemic model of non-proliferative diabetic retinopathy. Dis Model Mech.

[78] Cho NC, Poulsen GL, Ver Hoeve JN, Nork TM (2000). Selective loss of S-cones in diabetic retinopathy. Arch Ophthalmol.

[79] McAnany JJ, Park JC (2019). Cone photoreceptor dysfunction in early-stage diabetic retinopathy: association between the activation phase of cone phototransduction and the flicker electroretinogram. Invest Ophthalmol Vis Sci.

[80] Di R, Luo Q, Mathew D, Bhatwadekar AD (2019). Diabetes alters diurnal rhythm of electroretinogram in db/db mice. Yale J Biol Med.

[81] Samuels IS, Bell BA, Pereira A, Saxon J, Peachey NS (2015). Early retinal pigment epithelium dysfunction is concomitant with hyperglycemia in mouse models of type 1 and type 2 diabetes. J Neurophysiol.

[82] McDowell RE, McGahon MK, Augustine J, Chen M, McGeown JG, Curtis TM (2016). Diabetes impairs the aldehyde detoxifying capacity of the retina. Invest Ophthalmol Vis Sci.

[83] Pan HZ, Zhang H, Chang D, Li H, Sui H (2008). The change of oxidative stress products in diabetes mellitus and diabetic retinopathy. Br J Ophthalmol.

[84] Augustine J, Troendle EP, Barabas P, McAleese CA, Friedel T, Stitt AW (2020). The role of lipoxidation in the pathogenesis of diabetic retinopathy. Front Endocrinol (Lausanne).

[85] Jennings CL, Saadane A, Leinonen H, Elliot HC, Gao F, Lewandowski D (2023). Stress resilience-enhancing drugs preserve tissue structure and function in degenerating retina via phosphodiesterase inhibition. Proc Natl Acad Sci USA.

[86] Van Hove I, De Groef L, Boeckx B, Modave E, Hu TT, Beets K (2020). Single-cell transcriptome analysis of the Akimba mouse retina reveals cell-type-specific insights into the pathobiology of diabetic retinopathy. Diabetologia.

[87] Ly A, Scheerer MF, Zukunft S, Muschet C, Merl J, Adamski J (2014). Retinal proteome alterations in a mouse model of type 2 diabetes. Diabetologia.

[88] Kandpal RP, Rajasimha HK, Brooks MJ, Nellissery J, Wan J, Qian J (2012). Transcriptome analysis using next generation sequencing reveals molecular signatures of diabetic retinopathy and efficacy of candidate drugs. Mol Vis.

[89] Grant MB, Afzal A, Spoerri P, Pan H, Shaw LC, Mames RN (2004). The role of growth factors in the pathogenesis of diabetic retinopathy. Expert Opin Investig Drugs.

[90] Kjell J, Fischer-Sternjak J, Thompson AJ, Friess C, Sticco MJ, Salinas F (2020). Defining the adult neural stem cell niche proteome identifies key regulators of adult neurogenesis. Cell Stem Cell.

[91] Vogel C, Marcotte EM (2012). Insights into the regulation of protein abundance from proteomic and transcriptomic analyses. Nat Rev Genet.

[92] Liu Y, Beyer A, Aebersold R (2016). On the dependency of cellular protein levels on mRNA abundance. Cell.

[93] Imai S, Otsuka T, Naito A, Shimazawa M, Hara H (2017). Triamcinolone acetonide suppresses inflammation and facilitates vascular barrier function in human retinal microvascular endothelial cells. Curr Neurovasc Res.

[94] Stewart EA, Saker S, Amoaku WM (2016). Dexamethasone reverses the effects of high glucose on human retinal endothelial cell permeability and proliferation in vitro. Exp Eye Res.

[95] Sulaiman RS, Kadmiel M, Cidlowski JA (2018). Glucocorticoid receptor signaling in the eye. Steroids.

[96] Wenzel A, Grimm C, Samardzija M, Reme CE (2003). The genetic modifier Rpe65Leu(450): effect on light damage susceptibility in c-Fos-deficient mice. Invest Ophthalmol Vis Sci.

[97] Wenzel A, Grimm C, Seeliger MW, Jaissle G, Hafezi F, Kretschmer R (2001). Prevention of photoreceptor apoptosis by activation of the glucocorticoid receptor. Invest Ophthalmol Vis Sci.

[98] Zhang X, Lai D, Bao S, Hambly BD, Gillies MC (2013). Triamcinolone acetonide inhibits p38MAPK activation and neuronal apoptosis in early diabetic retinopathy. Curr Mol Med.

[99] Gjerstad JK, Lightman SL, Spiga F (2018). Role of glucocorticoid negative feedback in the regulation of HPA axis pulsatility. Stress.

[100] Erickson RL, Browne CA, Lucki I (2017). Hair corticosterone measurement in mouse models of type 1 and type 2 diabetes mellitus. Physiol Behav.

[101] Kadmiel M, Ramamoorthy S, Cidlowski J (2015). Glucocorticoid receptor role in the mouse retina. Investig Ophthalmol Vis Sci..

[102] Ghaseminejad F, Kaplan L, Pfaller AM, Hauck SM, Grosche A (2020). The role of Muller cell glucocorticoid signaling in diabetic retinopathy. Graefes Arch Clin Exp Ophthalmol.

[103] Totaro A, Panciera T, Piccolo S (2018). YAP/TAZ upstream signals and downstream responses. Nat Cell Biol.

[104] Ma S, Meng Z, Chen R, Guan KL (2019). The hippo pathway: biology and pathophysiology. Annu Rev Biochem.

[105] Hamon A, Garcia-Garcia D, Ail D, Bitard J, Chesneau A, Dalkara D (2019). Linking YAP to muller glia quiescence exit in the degenerative retina. Cell Rep.

[106] Rueda EM, Hall BM, Hill MC, Swinton PG, Tong X, Martin JF (2019). The hippo pathway blocks mammalian retinal Muller glial cell reprogramming. Cell Rep.

[107] Kim SL, Choi HS, Kim JH, Lee DS (2020). The antiasthma medication ciclesonide suppresses breast cancer stem cells through inhibition of the glucocorticoid receptor signaling-dependent YAP pathway. Molecules.

[108] Sorrentino G, Ruggeri N, Zannini A, Ingallina E, Bertolio R, Marotta C (2017). Glucocorticoid receptor signalling activates YAP in breast cancer. Nat Commun.

[109] Peng C, Zhu Y, Zhang W, Liao Q, Chen Y, Zhao X (2017). Regulation of the Hippo-YAP Pathway by Glucose Sensor O-GlcNAcylation. Mol Cell.

[110] Zhang X, Qiao Y, Wu Q, Chen Y, Zou S, Liu X (2017). The essential role of YAP O-GlcNAcylation in high-glucose-stimulated liver tumorigenesis. Nat Commun.

[111] Wu Y, Pan Q, Yan H, Zhang K, Guo X, Xu Z (2018). Novel mechanism of foxo1 phosphorylation in glucagon signaling in control of glucose homeostasis. Diabetes.

[112] Yang Z, Alvarez BV, Chakarova C, Jiang L, Karan G, Frederick JM (2005). Mutant carbonic anhydrase 4 impairs pH regulation and causes retinal photoreceptor degeneration. Hum Mol Genet.

[113] Wong TY, Cheung CM, Larsen M, Sharma S, Simo R (2016). Diabetic retinopathy. Nat Rev Dis Primers.

[114] Arjomandnejad M, Dasgupta I, Flotte TR, Keeler AM (2023). Immunogenicity of recombinant adeno-associated virus (AAV) vectors for gene transfer. BioDrugs.

[115] Verdera HC, Kuranda K, Mingozzi F (2020). AAV vector immunogenicity in humans: a long journey to successful gene transfer. Mol Ther.

[116] Scholz R, Caramoy A, Bhuckory MB, Rashid K, Chen M, Xu H (2015). Targeting translocator protein (18 kDa) (TSPO) dampens pro-inflammatory microglia reactivity in the retina and protects from degeneration. J Neuroinflammation.

[117] Mages K, Grassmann F, Jagle H, Rupprecht R, Weber BHF, Hauck SM (2019). The agonistic TSPO ligand XBD173 attenuates the glial response thereby protecting inner retinal neurons in a murine model of retinal ischemia. J Neuroinflammation.

[118] Wang M, Wang X, Zhao L, Ma W, Rodriguez IR, Fariss RN (2014). Macroglia-microglia interactions via TSPO signaling regulates microglial activation in the mouse retina. J Neurosci.

[119] Schmalen A, Lorenz L, Grosche A, Pauly D, Deeg CA, Hauck SM (2021). Proteomic phenotyping of stimulated muller cells uncovers profound pro-inflammatory signaling and antigen-presenting capacity. Front Pharmacol.

[120] Kaczmarek-Hajek K, Zhang J, Kopp R, Grosche A, Rissiek B, Saul A, et al. Re-evaluation of neuronal P2X7 expression using novel mouse models and a P2X7-specific nanobody. Elife. 2018;7. 10.7554/eLife.36217 PMC6140716 30074479

[121] Niu T, Fang J, Shi X, Zhao M, Xing X, Wang Y (2021). Pathogenesis study based on high-throughput single-cell sequencing analysis reveals novel transcriptional landscape and heterogeneity of retinal cells in type 2 diabetic mice. Diabetes.

[122] Peng BY, Wang Q, Luo YH, He JF, Tan T, Zhu H (2018). A novel and quick PCR-based method to genotype mice with a leptin receptor mutation (db/db mice). Acta Pharmacol Sin.

[123] Slezak M, Grosche A, Niemiec A, Tanimoto N, Pannicke T, Munch TA (2012). Relevance of exocytotic glutamate release from retinal glia. Neuron.

[124] Uckermann O, Iandiev I, Francke M, Franze K, Grosche J, Wolf S (2004). Selective staining by vital dyes of Muller glial cells in retinal wholemounts. Glia.

[125] Schindelin J, Arganda-Carreras I, Frise E, Kaynig V, Longair M, Pietzsch T (2012). Fiji: an open-source platform for biological-image analysis. Nat Methods.

[126] Chou JC, Rollins SD, Fawzi AA (2013). Trypsin digest protocol to analyze the retinal vasculature of a mouse model. J Vis Exp.

[127] Brandl C, Grassmann F, Riolfi J, Weber BH (2015). Tapping stem cells to target AMD: challenges and prospects. J Clin Med.

[128] Grassmann F (2019). Conduct and quality control of differential gene expression analysis using high-throughput transcriptome sequencing (RNASeq). Methods Mol Biol.

[129] Kechin A, Boyarskikh U, Kel A, Filipenko M (2017). cutPrimers: a new tool for accurate cutting of primers from reads of targeted next generation sequencing. J Comput Biol.

[130] Kim D, Langmead B, Salzberg SL (2015). HISAT: a fast spliced aligner with low memory requirements. Nat Methods.

[131] Corces MR, Trevino AE, Hamilton EG, Greenside PG, Sinnott-Armstrong NA, Vesuna S (2017). An improved ATAC-seq protocol reduces background and enables interrogation of frozen tissues. Nat Methods.

[132] Lun AT, Smyth GK (2016). csaw: a Bioconductor package for differential binding analysis of ChIP-seq data using sliding windows. Nucleic Acids Res.

[133] Machlab D, Burger L, Soneson C, Rijli FM, Schubeler D, Stadler MB (2022). monaLisa: an R/Bioconductor package for identifying regulatory motifs. Bioinformatics.

[134] Frik J, Merl-Pham J, Plesnila N, Mattugini N, Kjell J, Kraska J, et al. Cross-talk between monocyte invasion and astrocyte proliferation regulates scarring in brain injury. EMBO Rep. 2018;19(5). 10.15252/embr.201745294 PMC5934774 29632244

[135] Perez-Riverol Y, Bai J, Bandla C, Garcia-Seisdedos D, Hewapathirana S, Kamatchinathan S (2022). The PRIDE database resources in 2022: a hub for mass spectrometry-based proteomics evidences. Nucleic Acids Res.

