# Supplementary material for: The glucocorticoid receptor as a master regulator of the Müller cell response to diabetic conditions in mice
Source: J Neuroinflammation. 2024 Jan 25;21:33. doi: 10.1186/s12974-024-03021-x (PMC10809506; doi:10.1186/s12974-024-03021-x)
Supplement: Supplementary file 1 — Additional file 1. Supplementary information provides more extensive description details of various studies on the used db/db mouse strain, focusing on the validation of vascular alterations (Fig. S1), validadtion of MACS-based enrichment effectiveness and glial activation analysis using RNAseq and mass spectrometry (Fig. S2), transcriptome and proteome comparison in Müller cells from diabetic retinas, highlighting concordant expression patterns (Fig. S3), unprocessed Western blots for GR and PDHB analysis in different cell types and conditions (Fig. S4) and a phenotypic comparison regarding body weight and ERG recordings of db/db mice from different laboratory backgrounds. [file 12974_2024_3021_MOESM1_ESM.docx]

Additional file 1

**The glucocorticoid receptor as a master regulator of the Müller cell response to diabetic conditions in mice**

Anna M. Pfaller^1^, Lew Kaplan^1^, Madalena Carido^2^, Felix Grassmann^3,4^, Nundehui Díaz-Lezama ^1,5^, Farhad Ghaseminejad^1^, Kirsten A. Wunderlich^1,6^, Sarah Glänzer^1^, Oliver Bludau^1^, Thomas Pannicke^7^, Bernhard H.F. Weber^3,8^, Susanne F. Koch^1,5^, Boyan Bonev^1,2^, Stefanie M. Hauck^9^, Antje Grosche^1^

^1^ Physiological Genomics, Biomedical Center, Ludwig-Maximilians-Universität München, Germany

^2^ Helmholtz Pioneer Campus, Helmholtz Zentrum München, German Research Center for Environmental Health, Neuherberg, Germany

^3^ Institute of Clinical Human Genetics, University Hospital Regensburg, Regensburg, Germany

^4^ Institute for Clinical Research and Systems Medicine, Health and Medical University, Potsdam, Germany

^5^ Department of Pharmacy, Center for Drug Research, Ludwig-Maximilians-Universität München, Munich, Germany.

^6^ Institute for Molecular Medicine, Health and Medical University, Potsdam, Germany

^7^ Paul Flechsig Institute for Brain Research, University of Leipzig, Leipzig, Germany

^8^ Institute of Human Genetics, University Regensburg, Regensburg, Germany

^9^ Metabolomics and Proteomics Core, Helmholtz Zentrum München, German Research Center for Environmental Health, Neuherberg, Germany

*
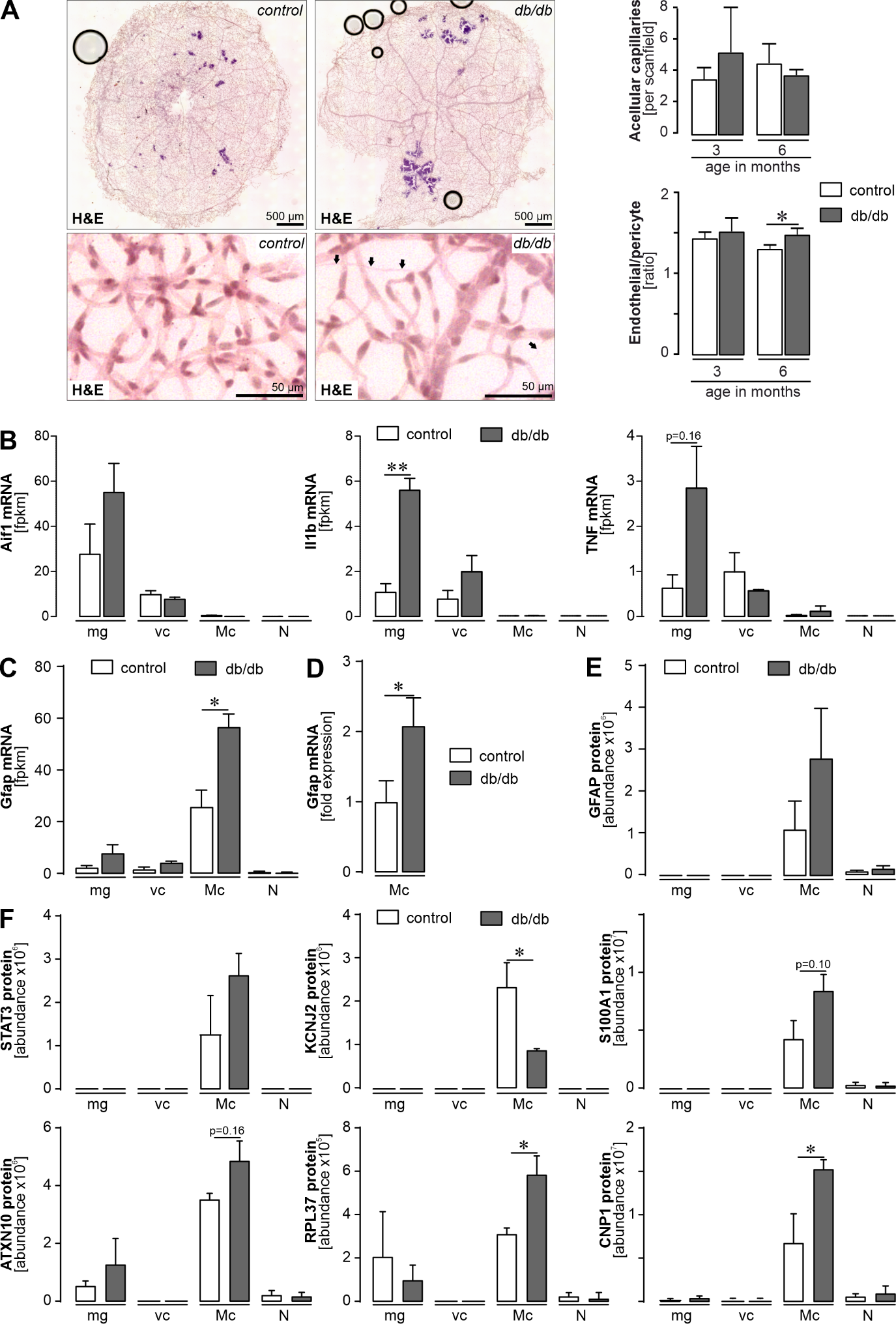
*

*Figure S1. Validation of the vascular alterations in the db/db mouse retina. Six-month-old db/db mice recapitulate some important vascular features of DR.* Left*, Representative images of hematoxylin and eosin (H&E) staining of trypsin-digested retinae from diabetic and control mice at 6 months of age. The arrows indicate acellular capillaries.* Right*, the number of acellular capillaries, endothelial cells, and pericytes per scan field was quantified in trypsin-digested flatmounts from db/db and control animals at 3 and 6 months of age. The ratio of endothelial cells to pericytes in the retina of db/db and control animals was then calculated using the respective cell counts. Bars represent mean ± SEM with the following number of biological replicates per age and genotype: 3 months of age, n=3 animals per genotype; 6 months of age, n=4 animals per genotype. Unpaired t-test: *P<0.05. Scan field: 100.05 µm x 100.05 µm.*

*
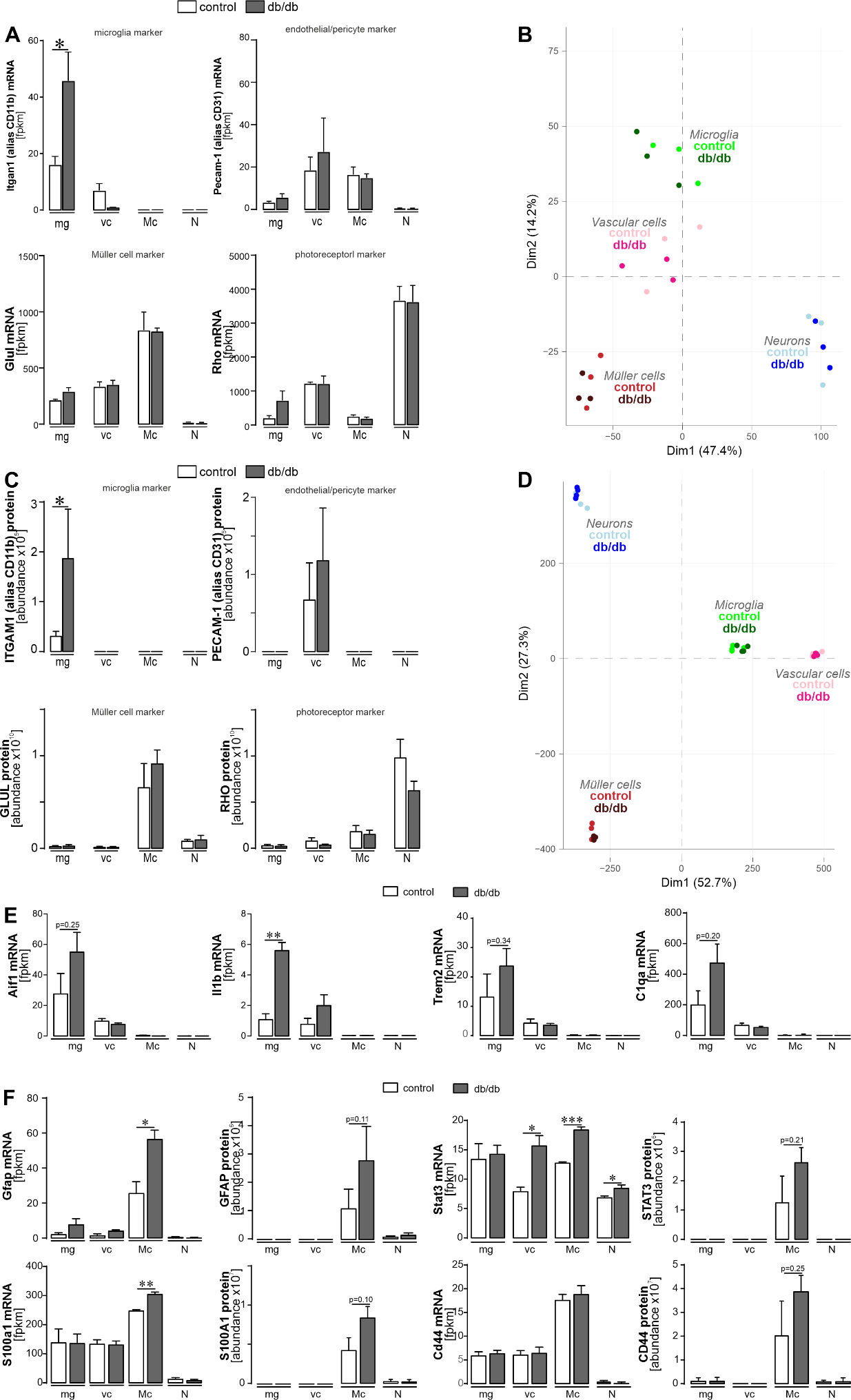
*

*Figure S2: Validation of efficiency of MACS-based enrichment of retinal cell populations isolated from 6-month-old diabetic and control animals and onset of glial activation by RNA-seq data and tandem mass spectrometry.*

*(A) Plotting of mRNA expression levels of established cell markers as determined by RNA-seq demonstrates successful enrichment of retinal cell types from 6-month-old mice by magnetic activated cell sorting (MACS) irrespective of the genotype. Itgam, integrin subunit alpha M; Pecam1, platelet and endothelial cell adhesion molecule 1; Glul, glutamine synthetase; rho, rhodopsin. Bars represent mean ± SEM (cells purified from n=3 animals per genotype). Unpaired t-test: *P<0.05.*

*(B) Principal component analysis (PCA) of the RNA-seq data from major retinal cell types enriched from retinae of control and diabetic mice at 6 months of age. Distinct clusters are formed by the different cell types implicating a good level of cell enrichment, while age and genotype seem to be less relevant for cluster formation.*

*(C) The protein expression of marker genes for the four cell types implicated a successful separation of the different cell population from 6-month-old mice. GLUL: Glutamine synthetase. RHO, rhodopsin; ITGAM, integrin subunit alpha M; PECAM1, platelet and endothelial cell adhesion molecule 1. Bars represent mean ± SEM (n=4 mice per genotype).*

*(D) Principal component analysis (PCA) of the proteomics data from four diabetic and control mice with an age of 6 month. Distinct clusters are formed by the four cell type groups.*

*(E) Transcript levels as determined by RNA-seq of known marker genes of microglial activation are plotted for retinal cell types purified via magnetic activated cell sorting. Bars represent mean ± SEM (n=3 mice per genotype). Unpaired t-test **P<0.01.*

*(F) Transcript levels as determined by RNA-seq and protein abundances as determined by MS/MS mass spectrometry of known marker genes of Müller cell gliosis are plotted comparing them with the different MACS-purified retinal cell types isolated from retinae of 6-month-old mice. Bars represent mean ± SEM (n=4 animals per genotype). Unpaired t-test *P<0.05; **P<0.01; ***P<0.001*

*(A, C, E, F) mg, microglia; vc, vascular cells; Mc, Müller cells; n, neurons.*

*
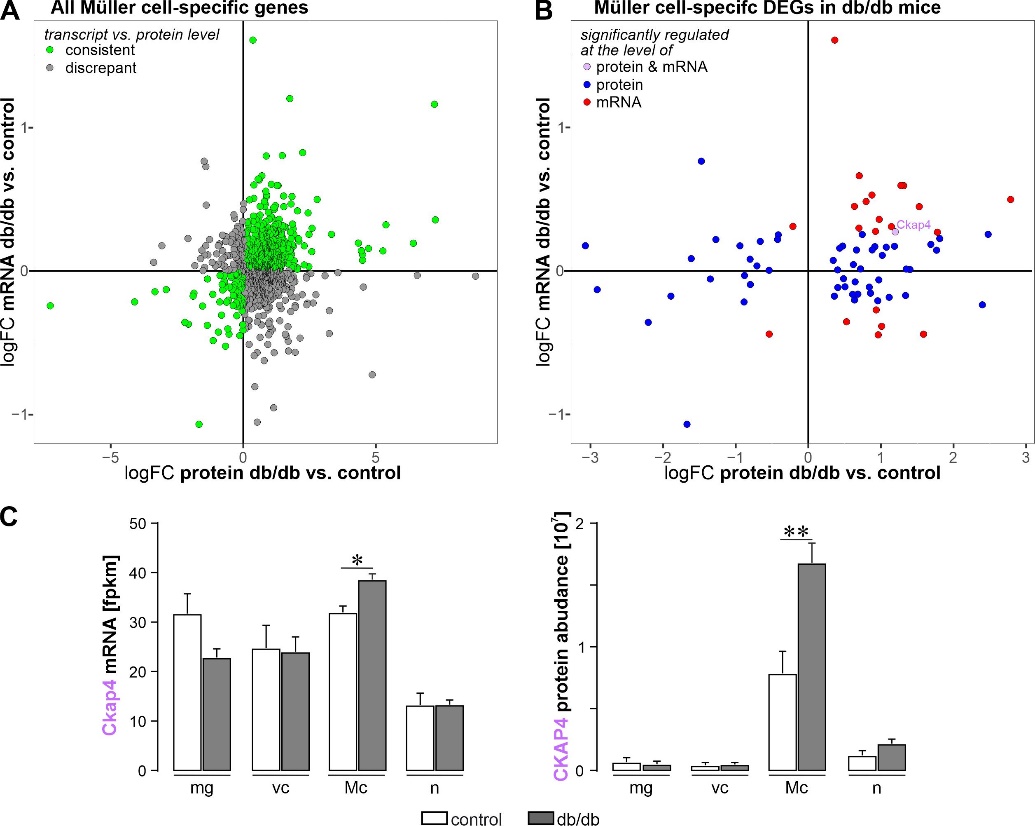
*

*Figure S3. Comparison of Müller cell-specific genes in the diabetic retina at the transcriptome and proteome levels.*

*(A) Comparison of the results of RNA-seq and proteome analysis focusing on genes that are Müller cell-specific at either protein or mRNA level shows that out of the 883 genes consistently present in both data sets, 56.4% of the genes show a concordant expression pattern at the transcript and protein level.*

*(B) Considering only the Müller cell-specific genes that additionally show significant differential expression in db/db mice at 6 months of age, 57.3% of the 75 genes show a concordant regulation pattern at transcript and protein level.*

*(C) MRNA and protein abundance of CKAP4 in microglia (mg), vascular cells (vc), Müller cells (Mc) and retinal neurons (n) as determined by RNA-seq and MS/MS mass spectrometry. Bars represent mean ± SEM (n=3 mice per genotype for RNA-seq data; n=4 mice per genotype for proteome analysis). Unpaired t-test: *P<0.5; **P<0.01.*

*
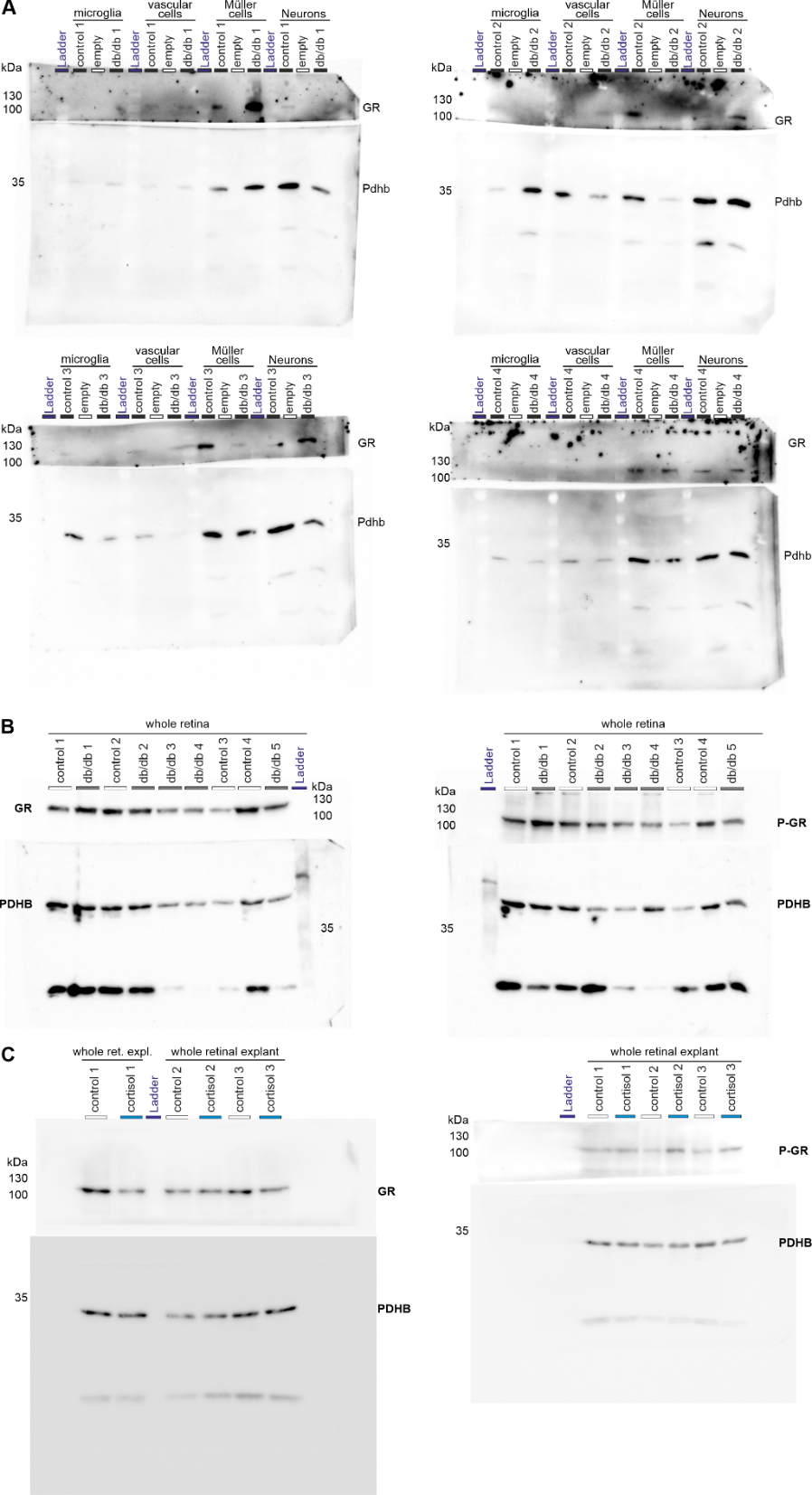
*

*Figure S4. Overview of all uncropped Western blots used for the analysis shown in Figures 5 and 6.*

1. *Western blots for the analysis shown in Figure 5B. Each blot was cut at the 70 kDa band of the ladder to detect the high molecular weight GR (95 kDa) and the low molecular weight housekeeper PDHB (~34 kDa) on the same blot. Since the amount of protein per MACS-purified cell population was very low, the amount of protein loaded per lane was not adjusted (the whole extract per cell population was loaded), but normalized to the housekeeper expression.*
2. *Western blots for the analysis shown in Figure 5D. Each blot was cut at the 70 kDa band of the ladder to detect GR (95 kDa) or its phosphorylated form and the housekeeper PDHB (~34 kDa) on the same blot. 50 µg of protein extract from whole retinae was loaded per lane and expression levels were normalized to that of the housekeeper.*
3. *Western blots for the analysis shown in Figure 6B. Each blot was cut at the 70 kDa band of the ladder to detect GR (95 kDa) or its phosphorylated form and the housekeeper PDHB (~34 kDa) on the same blot. 50 µg of protein extract per retinal explant culture was loaded per lane and expression levels were normalized to that of the housekeeper.*

*
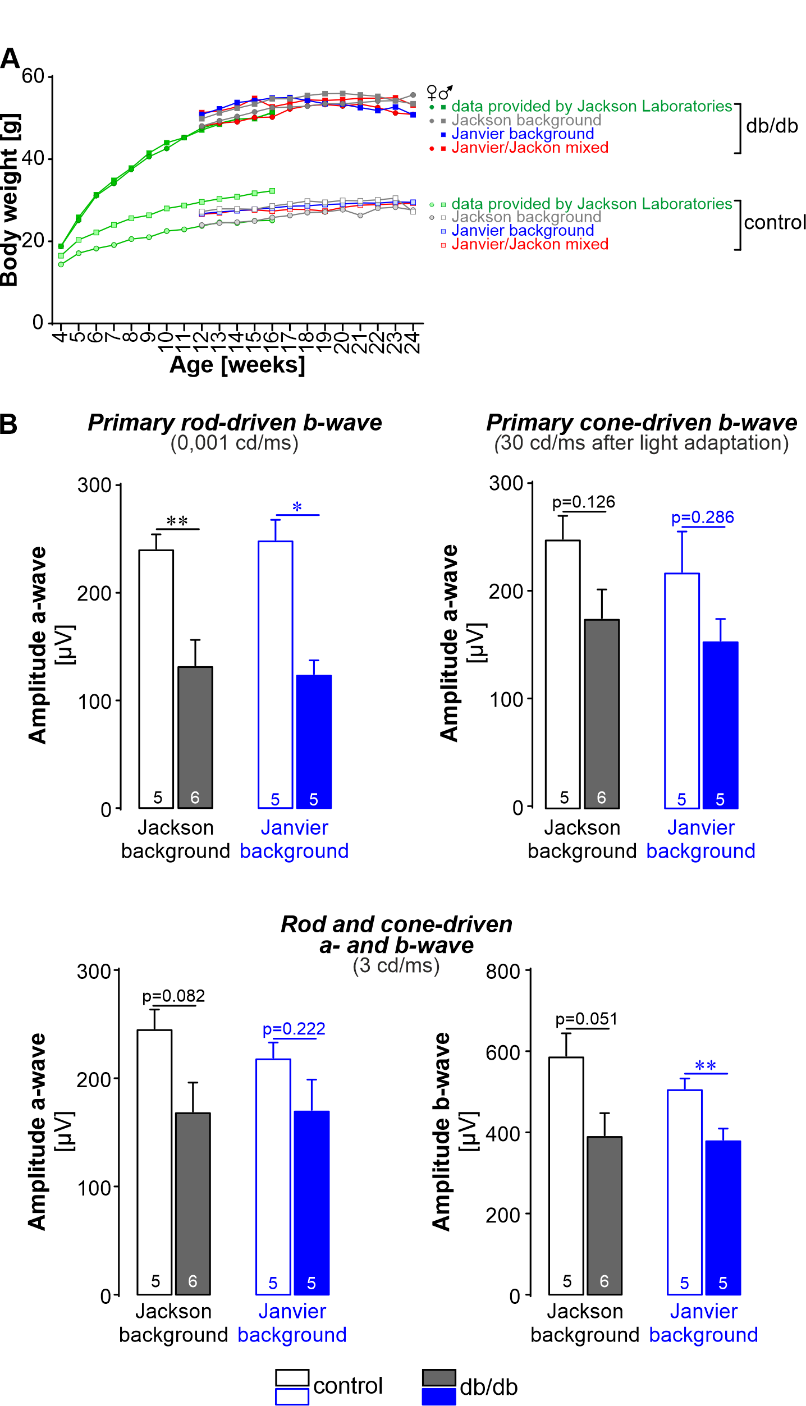
*

*Fig. S5. Phenotypic comparison of db/db mice from Janvier laboratories (BKS-Leprdb/db/JOrlRj) with those from Jackson laboratories (https://www.jax.org/strain/000642) and on a mixed Jackson/Janvier background.*

*(A) The body weight of mice from our db/db colony bred on a pure Jackson background or on a mixed Jackson/Janvier background with the indicated genotype and genetic background was plotted and compared to mice from Janvier (e.g. on a pure Janvier background). For comparison, we plotted the mean body weight of BKS.Cg-Dock7m+/+Leprdb/J mice as provided on the Jackson Laboratories website (*[*https://www.jax.org/strain/000642*](https://www.jax.org/strain/000642)*. Data from the following set of animals from our breeding colony are plotted: n=3 male control mice with Jackson background, n=5 female control mice with Jackson background, n=10 male db/db mice with Jackson background, n=10 female db/db mice with Jackson background, n=9 male control mice with Janvier background, n=5 male db/db mice with Janvier background, n=5 male control mice with mixed background, n=7 male db/db mice with mixed background, n=4 female db/db mice with mixed background.*

*(B)* *ERG recordings from mice from our db/db colony bred on a pure Mixed background or from animals ordered from Janvier (e.g. on a pure Janvier background). Data are plotted from 5-6 animals per genotype and genetic background, as indicated by the numbers within the bars.*
